# Long-term potentiation in spinal nociceptive pathways as a novel target for pain therapy

**DOI:** 10.1186/1744-8069-7-20

**Published:** 2011-03-28

**Authors:** Ruth Ruscheweyh, Oliver Wilder-Smith, Ruth Drdla, Xian-Guo Liu, Jürgen Sandkühler

**Affiliations:** 1 Department of Neurology, University of Münster, Münster, Germany; 2 Department of Anaesthesiology, Pain and Palliative Medicine, Radboud University Nijmegen Medical Centre, Nijmegen, The Netherlands; 3 Department of Neurophysiology, Center for Brain Research, Medical University of Vienna, Vienna, Austria; 4 Pain Research Center and Department of Physiology, Zhongshan School of Medicine, Sun Yat-sen University, Guangzhou, China

## Abstract

Long-term potentiation (LTP) in nociceptive spinal pathways shares several features with hyperalgesia and has been proposed to be a cellular mechanism of pain amplification in acute and chronic pain states. Spinal LTP is typically induced by noxious input and has therefore been hypothesized to contribute to acute postoperative pain and to forms of chronic pain that develop from an initial painful event, peripheral inflammation or neuropathy. Under this assumption, preventing LTP induction may help to prevent the development of exaggerated postoperative pain and reversing established LTP may help to treat patients who have an LTP component to their chronic pain. Spinal LTP is also induced by abrupt opioid withdrawal, making it a possible mechanism of some forms of opioid-induced hyperalgesia. Here, we give an overview of targets for preventing LTP induction and modifying established LTP as identified in animal studies. We discuss which of the various symptoms of human experimental and clinical pain may be manifestations of spinal LTP, review the pharmacology of these possible human LTP manifestations and compare it to the pharmacology of spinal LTP in rodents.

## Introduction

Pain arising from impending or actual tissue injury has an important physiological role, protecting the body from injury and promoting healing once injury has occurred. Pain persisting in the absence of ongoing nociceptive input from the periphery, or exceeding the pain normally caused by ongoing nociceptive input, has lost its physiological function and is therefore called maladaptive or dysfunctional [1]. Dysfunctional pain is thought to arise from altered processing of nociceptive information in the central nervous system.

One of the symptoms of clinically relevant pain is hyperalgesia, i.e. increased pain perception in response to painful stimuli [1,2]. This implies the presence of a mechanism that amplifies nociceptive excitation somewhere along the central nociceptive system. A synaptic amplifier of nociception has been identified at the synapses between primary afferent C-fibres, many of which are nociceptive, and neurons in the superficial dorsal horn of the spinal cord in rodents [3,4]. Amplification of nociceptive signals at this site can be "switched on" by noxious stimulation ("conditioning stimulation") of the associated nociceptive primary afferents. The underlying cellular mechanism is long-term potentiation (LTP) of synaptic strength, a mechanism also described in cortical regions like the hippocampus where it is thought to be the basis of memory formation [5].

Therefore, LTP at the first nociceptive synapse is currently regarded as a cellular model of hyperalgesia induced by noxious stimulation. As general anaesthesia without additional analgesia is not sufficient to protect the spinal cord from intraoperative noxious input [6,7], LTP in spinal nociceptive pathways may heighten acute postoperative pain. Moreover, in many patients with chronic dysfunctional pain, pain started to develop following an initial strong noxious input. Examples are chronic postoperative pain following intraoperative noxious input, chronic back pain developing from acute lumbago or sciatica and persistent idiopathic facial pain following major dental treatment [8-10]. Although there is currently no direct proof of the role of spinal LTP in human acute postoperative or chronic pain, some arguments have accumulated in favour:

(1) In rodents, LTP can be induced not only by electrical stimulation of primary afferents, but also by natural noxious stimulation, e.g. by peripheral inflammation and nerve injury [4,11-13].

(2) The same conditioning stimulation that induces LTP also leads to long-lasting hyperalgesia in freely behaving rodents [14,15].

(3) In rodents, LTP is preferentially expressed at synapses between nociceptive primary afferents and neurokinin 1 (NK1) receptor expressing projection neurons in lamina I, i.e. neurons that (a) relay nociceptive information directly to the brain and (b) have been shown to be necessary for the development of chronic pain [4,16-18].

(4) In rodents, the pharmacology of the induction of LTP is very similar to the pharmacology of induction of long-lasting hyperalgesia by models of chronic pain (inflammation, nerve injury), i.e. drugs that block LTP induction also block hyperalgesia induction (Table 2).

(5) Conditioning electrical stimulation of the same type that induces LTP in rodents has been shown to induce long-lasting potentiation of pain perception in humans [19,20].

In addition, is has recently been discovered that LTP at synapses between C-fibres and superficial dorsal horn neurons can also be induced by abrupt withdrawal of opioids [21]. Amplification of nociceptive information by LTP may therefore not only contribute to human hyperalgesia following an initial painful event but also to the clinically important phenomenon of hyperalgesia following opioid withdrawal [22].

A critical point regarding the significance of spinal LTP for long-lasting and chronic pain is its duration. In the hippocampus and other cortical regions, LTP may last between a few hours and the lifetime of the animal, depending upon the conditioning stimulus, its repetition and the experimental conditions [23,24]. Duration of LTP in spinal cord has not been studied directly. In one study, the hyperalgesia induced by LTP-inducing conditioning stimulation in healthy rodents reversed without further intervention after seven days [14]. In human volunteers, relatively mild conditioning stimulation causes hyperalgesia that lasts for about one day [25]. This time course seems consistent with a contribution of LTP to hyperalgesia following strong noxious stimulation, such as acute postoperative pain. In chronic pain patients, several factors may coincide to perpetuate LTP expression in nociceptive pathways, such as decreased activity of endogenous antinociceptive systems [13,26] or the presence of intermittent low-level nociceptive input from the periphery that might "boost" the maintenance of LTP, counteracting its natural reversal. Determining the factors influencing LTP duration beyond the first hours after induction will be important to understand the exact relationship between LTP and hyperalgesia in chronic pain patients.

In conclusion, LTP in spinal nociceptive pathways is in many respects an attractive model of short-term and possibly also long-term hyperalgesia and pain following noxious stimulation or opioid withdrawal. Preventing LTP induction, e.g. in the intraoperative setting, may prevent the development of exaggerated postoperative pain. Reversing established LTP may help to treat chronic pain patients who have an LTP component to their chronic pain.

In the present review, we first summarize current techniques for induction and recording of LTP in nociceptive pathways in rodents, then we give an overview of pharmacological and other possibilities to prevent the induction of LTP and disrupt the maintenance of established LTP in rodents. In the second part, potential manifestations of LTP in humans and the corresponding experimental and clinical models are discussed. Finally, the pharmacology of induction and maintenance of hyperalgesia in these human models is reviewed and compared to the pharmacology of LTP in rodents.

## Recording and induction of LTP in rodent spinal nociceptive pathways

### Recording of LTP in rodent spinal nociceptive pathways

LTP is defined as a long-lasting increase of synaptic strength [5] that can be mediated by either pre- or postsynaptic mechanisms, or both. Synaptic strength is the magnitude of the postsynaptic response (i.e. postsynaptic current or potential) in response to a single presynaptic action potential at a monosynaptic connection. Recording of LTP therefore has two prerequisites (1) investigation of a monosynaptic connection and (2) recording of postsynaptic currents or potentials. In the spinal cord, there are currently two methods to record synaptic strength in nociceptive pathways that fulfil the above requirements [2,27]. Both investigate the synaptic connection between primary afferent C-fibres (many of which are nociceptive) and superficial dorsal horn neurons, which is therefore the focus of the present review. *In vivo*, synaptic strength between primary afferent C-fibres and superficial dorsal horn neurons can be measured in adult rodents by stimulating the sciatic nerve and recording C-fibre-evoked field potentials in superficial dorsal horn that are known to reflect summation of postsynaptic, mainly monosynaptically evoked currents [3,28]. *In vitro*, spinal cord slice preparations from young rodents with long dorsal roots are most often used to selectively investigate the synapse between C-fibres and neurons with a known role in nociceptive processing, e.g. lamina I projection neurons that express the neurokinin 1 (NK1) receptor [4,17].

Several alternative methods have been used to investigate spinal LTP, but may not fulfil all of the above requirements. C-fibre evoked field potentials recorded in deep dorsal horn [14,29] are very similar to those recorded in superficial dorsal horn, but it is not clear if they reflect monosynaptic transmission from C-fibres. Action-potential firing recorded extracellularly from deep dorsal horn wide dynamic range (WDR) neurons [30,31] may in part reflect synaptic strength at the first nociceptive synapse but may also be affected by modifications of membrane excitability and synaptic inhibition. Optical imaging after bulk-loading of spinal cord slices with voltage-sensitive dyes does not allow distinction between neuronal and non-neuronal structures and between pre- and postsynaptic structures [4,32]. Where data from these studies is used in the text or tables, it is specifically indicated.

Voltage-sensitive dye can also be loaded into the presynaptic terminals of primary afferents over the dorsal root. This approach allows to selectively monitor presynaptic electrical activity, but the exact relationship to transmitter release is not known [32].

### Induction of LTP in rodent spinal nociceptive pathways

LTP at the synapse between primary afferent C-fibres and superficial dorsal horn neurons can be induced by various protocols, including strong noxious stimulation of the input pathway and application of certain drugs (Table 1). Most studies use noxious electrical stimulation of the dorsal root or sciatic nerve that can be exactly controlled regarding stimulus intensity and duration and is therefore highly reproducible. Both high frequency stimulation (HFS, several bursts at 100 Hz) and low frequency stimulation (LFS, 2 Hz for several min) of primary afferent C-fibres induce LTP at the first nociceptive synapse *in vivo *[3,4] and *in vitro *[4,17]. While HFS may reflect the discharge of a subtype of C-fibres at the beginning of noxious mechanical stimuli [33], LFS is similar to discharge rates of C-fibres during peripheral inflammation [34]. Indeed, LTP can also be induced by peripheral inflammation (injection of formalin into the hindpaw, [4]) and, after removal of descending inhibition, by noxious heat or mechanical stimulation of the skin [13]. Mechanical nerve injury is a frequently used animal model of neuropathic pain and also induces LTP [11,13]. A subset of primary afferent C-fibres express the transient receptor potential channel subfamily V member 1 (TRPV1) that is activated by both noxious heat and capsaicin and plays a major role in the induction of heat hyperalgesia [35]. Selective activation of these fibres by injection of capsaicin into the hindpaw has been shown to be sufficient for LTP induction [4], making TRPV1 antagonists or other methods that target the function of TRPV1-expressing C-fibres a potentially attractive target for prevention or modification of LTP at nociceptive spinal synapses. However, this has not been tested directly.

**Table 1:** Methods to induce LTP.

Type of stimulation		Protocol	*in vivo*	*in vitro*	Comments	References
Electrical nerve stimulation: C-fibres	HFS	100 Hz for 1 s, repeated 2-20 times at 10-20 s intervals	•	•		[3,4,6,17,40,65,70,90,92,101,102,108,110,113,133,134,143,144,268,269] (superficial dorsal horn), [14,29,114,270] (deep dorsal horn)
	LFS	2 Hz, 120 s	•	•		[4,7]
		1-2 Hz, 40-100 s paired with postsynaptic depolarisation		•		[38,271]
	IFS	10 Hz for 1 s, repeated 12 times at 10 s		•		[83]
		20 Hz for 5 s, repeated 4 times at 10 s intervals	•			[3]
Electrical nerve stimulation: Aδ-fibres	HFS	100 Hz for 1 s, repeated 90 times at 10 s intervals	•		LTP only in spinalised rats	[36]

Natural noxious stimulation		Noxious heat, pinching (hindpaw)	•		LTP only in spinalised rats	[13]
		Formalin, capsaicin injection (hindpaw)	•			[4,13]
		Sciatic nerve transsection or crush	•			[11]
		Sural nerve crush	•		LTP only in spinalised rats	[13]

Pharmacological stimulation		NMDA, substance P, neurokinin A	•		LTP only in spinalised rats	[272]
		ATP	•			[122]
		BDNF, SKF 38393 (Dopamine receptor D1/D5 agonist), 8-Br-cAMP (PKA activator)	•		Late, protein-synthesis- dependent phase of LTP	[91,140]
		Abrupt withdrawal of remifentanil or DAMGO	•	•	No LTP upon tapered withdrawal	[21,42]
		TNF-α	•		LTP only in neuropathic animals	[111]

HFS, high frequency stimulation; IFS, intermediate frequency stimulation, LFS, low frequency stimulation

LTP at the synapse between primary afferent C-fibres and superficial dorsal horn neurons can also be induced by manipulations not directly activating the input pathway. In spinalized animals, prolonged burst stimulation of primary afferent Aδ-fibres induces LTP of C-fibre-evoked field potentials, possibly reflecting heterosynaptic potentiation [36]. LTP can also be induced in the absence of presynaptic activity by application of certain drugs (Table 1). Of special interest may be the induction of LTP by abrupt opioid withdrawal that may represent a cellular mechanism of opioid-induced hyperalgesia [21].

## Modulation of spinal LTP in rodents by drugs and counterirritation

### Prevention of spinal LTP induction in rodents

Intracellular Ca^2+ ^rise in the postsynaptic neuron is a central step in the induction of many forms of LTP [5,37], including LTP in spinal dorsal horn [4,17,21,38]. When spinal LTP is induced by HFS or LFS, the massive release of glutamate from nociceptive primary afferents is thought to induce a postsynaptic depolarisation (primarily via α-amino-3-hydroxy-5-methyl-4-isoxazolepropionic acid [AMPA] receptors) strong enough to remove the Mg^2+ ^block from the *N*-methyl-*D*-aspartate (NMDA) receptor. Ca^2+ ^influx through the NMDA receptor is one of the key signals that activates the intracellular machinery involved in LTP induction [2,27,39]. However, the postsynaptic Ca^2+ ^rise achieved by NMDA receptor activation alone seems to be insufficient to induce LTP, as several parallel pathways that increase intracellular Ca^2+ ^have been shown to be necessary for LTP induction (e.g., Ca^2+ ^influx through T-type voltage-gated Ca^2+ ^channels [VGCCs] and Ca^2+ ^release from intracellular stores, triggered by activation of NK1 receptors and metabotropic glutamate receptors of group I [mGluRIs], see [3,4,7,17,38,40]).

**Figure 1:**
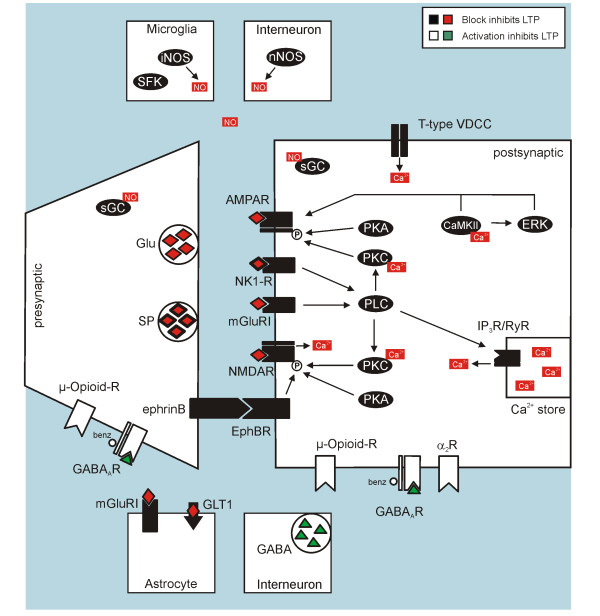
**Targets for prevention of spinal LTP induction in rodents**.

Therefore, LTP induction by conditioning stimulation can be interfered with at different stages: (1) Manipulations that reduce basal synaptic transmission at the first nociceptive synapse have the potential to prevent induction of LTP by indirectly preventing NMDA receptor activation. This is likely the case for μ-opioid-receptor antagonists (reduction of transmitter release and reduction of postsynaptic depolarization), AMPA receptor antagonists and γ-aminobutyric acid receptors of type A (GABA_A_R) agonists/current enhancers (prevention of postsynaptic depolarization) (2) Drugs that directly interfere with NMDA receptor activation (e.g. NMDA receptor antagonists, Xenon, possibly EphB receptor antagonists) (3) Drugs that interfere with additional sources of activity-dependent intracellular Ca^2+ ^rise (e.g. antagonists of T-type VGCCs, NK1 receptors or mGluRIs) (4) Drugs that interfere with intracellular pathways downstream from Ca^2+ ^influx (see section on signal transduction pathways). Targets for prevention of LTP induction are summarized in Table 2, illustrated in Figure 1 and are discussed below. Table 2 also shows that the pharmacology of prevention of LTP induction is equivalent to the pharmacology of the prevention of hyperalgesia induction in animal models of inflammation and neuropathic pain.

**Table 2:** Targets for prevention of LTP induction.

	Target	Substance	Action at target	HFS	LFS	Opioid with- drawal	*in vivo*	*in vitro*	Comments	References	Effect of equivalent drugs on hyperalgesia induction*
AMPAR	AMPAR	NBQX	antagonist	**X**			•		WDR neuron AP firing	[30]	**X **[273]

NMDAR	NMDAR	AP5, D-AP5, MK 801, ketamine	antagonist	**X**	**X**	**X**	•	•	NMDAR antagonists also prevent LTP induced by nerve transsection [11], BDNF [140] and LTP of human pain perception [20]	[4,7,13,17,21,42,83,101,102,134,268,274]	**X **[275]
	NMDA-2B R	Ro 25-6981	antagonist	**X**			•		WDR neuron AP firing	[276,277]	**X **[276]

mGluRs	mGluRI	AIDA, 4-CPG	antagonist	**X**	**X**		•	•	The mGluR1 antagonist LY367385 reduces long-lasting facilitation of presynaptic excitation [32] (optical imaging)	[38,40]	**X **[278-280]
	mGluRII, III	EGLU, LY341495, MSOP	antagonist	**0**			•			[40]	

VGCC	T-type VDCC	mibefradil, Ni^2+^	antagonist	**X**	**X**		•	•		[4,7,17]	
	α_2_δ-subunit of VGCCs	gabapentin		**0**			•			[65]	**0 **[60,62]

NK1R	NK1R	RP67580, 703,606	antagonist	**X**	**X**		•	•		[3,4,7,17]	**X **[69,281]

GABA_A_R	GABA_A_R	diazepam	Current amplifier	**X**			•			[70]	

Opioid receptors	μ-opioid receptors	fentanyl, DAMGO	agonist	**X**	**X**		•	•	Drugs depress baseline responses. Fentanyl prevents LTP at low but not high doses	[6,83]	**X **[282]

Descending inhibition	α_2_- adrenergic receptors	clonidine	agonist	**X**			•			[90]	**X **[283] (human capsicin model)
	5-HT_3 _receptor	odansetron	antagonist	**X**			•		WDR neuron AP firing	[31]	
	D1/D5 dopamine receptor	SCH 23390	antagonist	**0**			•		Selectively blocks L-LTP but not E-LTP	[91]	

Anaesthetic gases		isoflurane, sevoflurane, urethane		**0**	**0**	**0**	•			[3,4,6,7,21] and others	
		Xenon		**X**			•			[92]	

Neurotrophins	TrkB receptor	K252a, TrkB- Fc	Trk inhibitor, BNDF scavenger	**0**	**0**		•		Blocks L-LTP after LFS	[140]	**X **[284]

EphR-ephrin signalling	EphB R	EphB1-Fc EphB2-Fc	antagonist	**X**			•			[101,102]	**X **[100,102,285,286]
	EphB R	ephrinB1-Fc	agonist	**0**			•			[101,102]	

NO-pathway	NOS	L-NMMA, L-NAME	inhibitor	**X**	**X**		•	•	[14,29]: deep dorsal horn. Induction of long-lasting facilitation of presynaptic electrical activity by LFS is reduced by blockers of nNOS and iNOS [32] (optical imaging)	[4,14]	**X **[14,287,288]
	extracellular NO	PTIO, hemoglobin	scavenger	**X**	**X**		•	•		[4,14]	**X **[289] (NMDA-induced hyperalgesia)
	sGC	ODQ, MD	inhibitor	**X**	**X**		•	•		[4,29]	
	mono-, poly ADPRT	nicotonamide, benzamide	inhibitor	**0**			•			[29]	

Signal transduction pathways	CaMKII	KN-93, AIP, NK-62	inhibitor	**X**	**X**	**0**	•	•		[4,7,21,143]	**X **[290]
	PKA	Rp-CPT-cAMPS	inhibitor	**X**			•			[143]	**X **[291,292] (hyperalgesia induced by i.th. CGRP/subcutaneous bee venom injury)
	PKC	Chelerythrine, Gö 6983, GF109203X	inhibitor	**X**	**X**	**X**	•	•		[4,7,21,143]	**X**
	PLC	U73122	inhibitor	**X**	**X**		•	•		[4,7,17]	**X **[293]
	IP3R	2-APB	inhibitor	**X**	**X**			•		[4,17]	
	RyR	Dantrolene, ryanodine	inhibitor	**X**	**X**	**X**	•			[7,21,108]	
	ERK	PD98059	inhibitor	**X**			•			[110]	**X **[294]
	JNK	SP600125	inhibitor	**0**			•		Same drugs prevent induction of LTP by TNF-α in neuropathic rats	[111]	**X **[295]
	p38 MAPK	SB203580	inhibitor	**0**			•			[111]	**X **[296,297]

Glial cells/neuroimmune mechanisms	Glial metabolism	fluorocitrate	inhibitor	**X**			•		Deep dorsal horn. Under fluorocitrate, HFS induces LTD. Also blocks induction of long-lasting potentiation of presynaptic electrical activity by LFS [32] (optical imaging)	[114]	**X **[298]
	Microglia metabolism	minocycline	inhibitor	**X**			•		Under minocycline, HFS induces LTD	[113]	**X **[299]
	Microglia SKF (Src-family kinases)	PP2, SU6656	inhibitor	**X**			•		HFS activates SFKs selectivey in microglia. Under SKF inhibitors, HFS induces LTD	[113]	**X **[124]
	GLT-1	DHK	inhibitor	**X**			•		Deep dorsal horn	[128]	**X **[300]
	TNF α receptor	TNF-α	agonist		**0**		•			[111]	**X **[301]
	TNF-α	TNF-α antibody	inhibitor					•	Optical imaging	[123]	**X **[302,303]
	IL-6	IL-6 antibody	inhibitor					•	after bulk loading of voltage-sensitive dye; LTP induced by αβmeATP **X**	[123]	**X **[304] mechanical hyperalgesia induced by fractalkine injection; [297]

**X**, complete block or significant inhibition of LTP induction (left part of the table) or hyperalgesia induction (right part of the table)

**0**, no effect on LTP induction

* Gives example of reports where spinal administration of drugs before induction of hyperalgesia prevented or significantly depressed or delayed the development of hyperalgesia in response to peripheral inflammation, nerve injury or LTP-inducing conditioning stimulation. Where other stimuli were used to induce hyperalgesia, this is indicated. For a more complete review of drugs influencing hyperalgesia and allodynia, see [2].

Synaptic strength between primary afferent C-fibres and superficial dorsal horn neurons can be modified bidirectionally, with LTP or long-term depression (LTD) being induced depending on modalities of stimulation and on the stimulated pathway [36]. For cortical synapses, it has been proposed that the quantitative level of the activity-dependent rise in postsynaptic Ca^2+ ^determines whether synaptic strength will increase or decrease. LTP is believed to occur with higher Ca^2+ ^elevations that activate protein kinases while LTD would occur at lower Ca^2+ ^elevations that activate protein phosphatases, possibly with a large "neutral" Ca^2+ ^range between both states, where neither LTP nor LTD is induced [37,41]. In spinal cord, this has not been tested directly. However, drugs that interfere with intracellular Ca^2+ ^levels, like mGluRI receptor antagonists, can convert spinal LTP into LTD when applied during conditioning stimulation [38], suggesting that Ca^2+ ^dependence of LTP vs. LTD may be similar in spinal cord and cortex.

In addition to conditioning stimulation, LTP between primary afferent C-fibres and superficial dorsal horn neurons can also be induced by abrupt opioid withdrawal [21]. It has been proposed that this novel form of LTP is induced postsynaptically, sharing mechanisms with stimulation-induced LTP, as it is abolished by preventing postsynaptic Ca^2+ ^rise and by blocking postsynaptic G-protein coupled receptors or postsynaptic NMDA receptors [21]. The pre- vs. postsynaptic expression of opioid withdrawal LTP is currently a matter of debate, see [42] and our eLetter commenting on this paper available on the journal's web site.

#### Glutamate receptors

The induction of nearly all forms of spinal LTP is blocked by application of NMDA receptor antagonists (Table 2). This makes Ca^2+ ^influx through the NMDA receptor and consequent activation of downstream Ca^2+ ^dependent signal transduction one of the central requirements for the induction of spinal LTP [2,27].

At normal resting potential levels, such as present during baseline synaptic transmission, glutamate that binds to the NMDA receptor may or may not induce Ca^2+ ^influx because, depending on its subunit composition [43], the NMDA receptor channel may be blocked by Mg^2+ ^ions [44]. During LFS or HFS, massive glutamate release followed by strong activation of AMPA receptors is thought to provide the postsynaptic depolarization necessary to remove the Mg^2+ ^block from the NMDA receptor channel and enable LTP induction. The role of AMPA receptors has not been tested directly in superficial dorsal horn LTP, but induction of long-lasting facilitation of action potential discharges in WDR neurons is reduced by submaximal block of AMPA receptors [30].

While most types of AMPA receptors are permeable only for Na^+^, AMPA receptors lacking the GluR2 subunit are in addition permeable for Ca^2+ ^[45]. Ca^2+^-permeable AMPA receptors have been found on superficial dorsal horn neurons, including NK1 receptor expressing projection neurons [46,47], making them potentially suited to play a prominent role in spinal LTP. However, it is currently not known whether Ca^2+ ^influx through Ca^2+^-permeable AMPA receptors contributes to spinal LTP under normal conditions. GluR2 knockout mice, where presumably all AMPA receptors are permeable to Ca^2+^, show enhanced spinal LTP that is independent of NMDA receptors [48], demonstrating that under these conditions, Ca^2+ ^influx through AMPA receptors can substitute for Ca^2+ ^influx through NMDA receptors.

Intracellular Ca^2+ ^rise may also be initiated by activation of metabotropic receptors, e.g. mGluRIs, which mobilize intracellular Ca^2+ ^from intracellular stores by activation of ryanodine- and inositol-1,4,5-trisphosphate (IP3) receptors via phospholipase C (PLC) [49]. Indeed, induction of spinal LTP requires activation of mGluRIs [38,40]. In contrast, inhibition of group II and III mGluRs, that do not couple to the PLC/IP3 pathway [50,51], does not affect spinal LTP [40]. mGluRIs are also present on astrocytes [52], where they are thought to be involved in long-lasting facilitation of electrical activity in primary afferent terminals via the release of nitric oxide (NO) [32].

#### Voltage-gated calcium channels (VGCCs)

The strong postsynaptic depolarization achieved during HFS or LFS leads to activation of VGCCs that may thus also contribute to the activity-dependent Ca^2+ ^rise necessary for LTP induction. VGCCs are present on both primary afferent C-fibres and superficial dorsal horn neurons [53,54], and can be classified according to their activation threshold (high- or low-voltage gated channels), their subunit composition and their pharmacology [55]. Low-threshold T-type VGCCs open below action potential threshold [56] and their expression in superficial dorsal horn neurons is associated with a steep rise of intracellular Ca^2+ ^during conditioning stimulation that is necessary for induction of spinal LTP [4,7,17].

The α_2_δ-subunit is an auxiliary subunit of high-threshold VGCCs [57,58] that has recently become a focus of interest as it is a target of gabapentin and pregabalin, drugs that are successfully used in the therapy of neuropathic pain [59]. Gabapentin has little effect on basal synaptic transmission or acute pain [60-64]. Consistently, gabapentin does not affect LTP induction [65]. Results are different for actions of gabapentin on established neuropathic or inflammatory pain and established LTP (see below).

#### Neurokinin-1 receptors (NK1 receptors)

Repetitive stimulation of nociceptive primary afferents such as during HFS or LFS releases substance P into the dorsal horn [66], activating NK1 receptors located primarily on projection neurons with cell bodies in lamina I, III and IV [67,68]. Block of spinal NK1 receptors attenuates the induction of thermal and mechanical hyperalgesia [69]. This effect seems to rely on NK1 receptor expressing lamina I neurons because ablation of these neurons reduces the expression of hyperalgesia following nerve lesion or chronic inflammation [16,18]. Consistently, NK1 receptor antagonists block LTP induction by HFS and LFS of primary afferent C-fibres both in field potential recordings *in vivo *[3,7] and in patch-clamp recordings from NK1 receptor expressing lamina I projection neurons *in vitro *[4,17].

It has been proposed that activation of NK1 receptors during HFS or LFS contributes to the intracellular Ca^2+ ^elevation necessary for the induction of LTP by (1) inducing Ca^2+ ^release from IP3-sensitive intracellular stores via activation of PLC and (2) by increasing Ca^2+ ^influx through NMDA receptors via receptor phosphorylation by PLC-activated protein kinase C (PKC) [7,17].

#### GABA and glycine receptors

GABA, acting on GABA_A _and GABA_B _receptors, and glycine, acting on glycine receptors, are the main inhibitory transmitters in spinal cord. Of the three receptor types mentioned, only the GABA_A _receptor has been studied in relation to spinal LTP, using application of benzodiazepines [70] that enhance the action of GABA at the GABA_A _receptor by increasing the frequency of receptor channel openings [71]. Application of benzodiazepines prevents LTP induction [70]. As benzodiazepines do not open the GABA_A _receptor channel in the absence of GABA [71], this means that there is ongoing or HFS-induced GABA release in spinal cord dorsal horn that is not sufficient to block LTP induction on its own but becomes sufficient when amplified by the action of benzodiazepines. GABA_A _receptors are present both on the central terminals of primary afferent C-fibres, decreasing transmitter release, and on nociceptive superficial dorsal horn neurons, inducing hyperpolarization and/or shunting excitatory currents [72]. It is currently not clear whether the block of LTP induction by benzodiazepines is primarily due to reduced transmitter release during conditioning stimulation or prevention of the strong postsynaptic depolarization necessary for removal of the Mg^2+ ^block of the NMDA receptor channel and subsequent LTP induction.

#### Opioid receptors

Opioids are the gold standard for treatment of moderate to severe pain, and spinal actions seem to have a prominent role in their analgesic effect [73]. Of the three major subtypes of opioid receptors, μ-, δ- and κ-receptors, μ-opioid receptors predominate in spinal dorsal horn and are present on both primary afferent C-fibres and excitatory superficial dorsal horn neurons [74,75]. κ- and δ-opioid receptors have also been identified on primary afferent fibres and/or superficial dorsal horn neurons [76-78].

Opioid receptors are mostly coupled to Gi/o proteins. Activation leads to inhibition of voltage-gated Ca^2+ ^channels, opening of G-protein coupled inwardly rectifying K^+^-channels (GIRKs) and inhibition of adenylyl cyclase [79]. These mechanisms decrease synaptic transmission and neuronal excitability of spinal neurons by both pre- and postsynaptic actions, i.e. by induction of hyperpolarisation, by inhibition of transmitter release from neuronal terminals and/or by interfering with intracellular protein kinases and gene transcription [80].

Most of the opioids in clinical use target the μ-opioid receptor. At the synapse between primary afferent C-fibres and spinal dorsal horn neurons, μ-opioid receptor agonists acutely inhibit synaptic transmission by a predominantly presynaptic mechanism involving inhibition of N- and P/Q-type VGCCs [21,81,82]. As described above, depression of basal synaptic transmission is able to interfere with LTP induction by conditioning stimulation. Indeed, μ-opioid receptor agonists block LTP induction when administered prior to conditioning stimulation both *in vitro *and *in vivo *[6,83]. Interestingly, *in vivo *this effect is present only at medium doses but not at high doses of i.v. fentanyl, possibly due to an activation of NMDA receptors by opioid receptor agonists [84]. μ-opioid receptor agonists may activate NMDA receptors either directly [85] or indirectly via activation of PKC or cAMP-dependent protein kinase (PKA) [86,87]. Although a strong depression of basal synaptic transmission by reducing presynaptic transmitter release should probably be sufficient to prevent LTP induction, it has not been tested directly which of the above described actions of μ-opioids are crucial in preventing spinal LTP. The effect of application of δ- or κ-opioid receptor agonists during induction of spinal LTP has not been studied so far.

#### Receptor systems targeted by descending pathways: Adrenergic, dopaminergic and serotonin receptors

Spinal nociception is subject to descending control from several brain regions, including midbrain periaqueductal gray (PAG), the nucleus locus coeruleus, the nucleus raphe magnus (NRM) and the rostral ventromedial medulla (RVM). Descending control can have both inhibitory and facilitatory effects on nociceptive spinal transmission and critically influences the pain experience in acute and chronic pain states [88]. The descending control systems exert their effects by releasing a variety of neurotransmitters and/or neuromodulators, such as norepinephrine, serotonin (5-hydroxytryptamine, 5-HT) and dopamine [89].

Removing descending control in deeply anaesthetized adult rats by spinalization leads to a potentiation of C-fibre evoked field potentials by up to 250% of control [36]. Prolonged burst stimulation of the sciatic nerve at Aδ-fibre strength produces LTD of C-fibre-evoked field potentials in intact rats but LTP in spinalized animals [36]. Similarly, spinalization facilitates LTP induction by natural noxious stimulation [13]. These results demonstrate that the descending control system has an overall tonic inhibitory effect on C-fibre-mediated synaptic transmission that counteracts LTP induction. Consistently, mimicking activation of descending inhibitory pathways by spinal application of the α_2_-adrenergic receptor agonist clonidine before HFS prevents LTP induction [90]. The effects of 5-HT or dopamine agonists on LTP induction have not been examined. Block of D1/D5 dopamine receptors does not affect LTP induction [91]. Block of the excitatory 5-HT_3 _receptor, hypothesized to be involved in descending facilitatory pathways, reduces the long-lasting increase in WDR neuron action potential firing induced by HFS [31].

#### Anaesthetic gases

Deep surgical levels of anaesthesia with either urethane, isoflurane or sevoflurane are insufficient to prevent LTP induction of C-fibre-evoked field potentials following HFS [6], LFS [4,7] or opioid withdrawal [21]*in vivo*. In contrast, the noble gas xenon, which has not only anaesthetic but also NMDA receptor blocking properties, prevents induction of LTP at C-fibre synapses in intact rats [92].

#### Neurotrophin receptors

Brain-derived neurotrophic factor (BDNF) is constitutively synthesized in a subpopulation of primary afferent C-fibres [93] and is released into the superficial layers of the spinal dorsal horn along with substance P and glutamate in an activity-dependent manner [94]. Its receptor TrkB, a tyrosine kinase, had been found on both primary afferents and superficial dorsal horn neurons [95]. BDNF is not necessary for induction of LTP [12] but has been reported to be involved in LTP maintenance and can induce LTP in the absence of primary afferent input (see Table 1 and below).

#### Transsynaptic Eph-ephrin interactions

The ephrins (ephrinA and B with subtypes) are membrane-bound presynaptic proteins that bind to postsynaptic Eph receptor tyrosine kinases (EphA and EphB receptors with subtypes), regulating dendritic spine formation and controlling synaptic organization by interaction with AMPA, NMDA and mGluR receptors [96-99]. Within the B subclass, which has been studied in spinal cord, ligand-receptor binding is not subtype-specific (e.g., ephrinB2 is able to activate the EphB1 receptor) [98].

EphrinB2 is present in small, nociceptive dorsal root ganglion neurons, and EphB1 receptors have been detected in superficial dorsal horn, suggesting that the transsynaptic Eph-ephrin interaction may be involved in spinal nociceptive processing [100]. Indeed, EphB-ephrinB signalling is necessary for both the induction of LTP by HFS and the induction of hyperalgesia in models of inflammatory and neuropathic pain [100-102]. It has been proposed that this is due to interactions between EphB receptors and NMDA receptors. Activated EphB receptors associate with synaptic NMDA receptors [96] and induce NMDA receptor phosphorylation, possibly via phosphorylation of the NR2B subunit involving Src kinase activity, thereby increasing Ca^2+ ^influx through the NMDA receptor [103,104]. Consistently, administration of exogenous EphB receptor activators lowers the threshold for LTP induction by electrical stimulation in an NMDA receptor dependent manner [102].

#### Nitric oxide (NO) pathway

The gaseous molecule NO is a cell-permeant neuromodulator that is synthesized on demand by the enzyme nitric oxide synthase (NOS) that exists in different isoforms (neuronal, endothelial, inducible: nNOS, eNOS, iNOS). NO-imaging has shown that NO is released into the dorsal horn by repetitive stimulation of nociceptive primary afferents such as during LFS [105]. Consistently, LTP induction by HFS and LFS is blocked when NO production is suppressed, when NO is prevented from crossing the extracellular space, or when the primary target of NO, soluble guanylyl cyclase (sGC), is inhibited [4,14,29]. In contrast, activation of ADP-ribosyltransferases (ADPRTs), an alternative intracellular target of NO, is not necessary for LTP induction [29]. As NOS is very scarce both in lamina I projection neurons and in primary afferents [106], NO seems to act neither as an anterograde nor as a retrograde transmitter at the first nociceptive synapse during LTP induction between primary afferent C-fibres and lamina I projection neurons. It has been proposed that NO is produced in neighboring interneurons, glial cells or blood vessels, crosses the extracellular space and acts in lamina I projection neurons (most of which express sGC) and/or nociceptive primary afferents (some of which also express sGC) [4,32,106].

#### Intracellular signal transduction pathways

Signal transduction pathways involved in spinal LTP are similar to those reported for hippocampal LTP [107]. Specifically, inhibitors of calcium/calmodulin-dependent protein kinase II (CaMKII), PKA, PKC and PLC all have been shown to prevent induction of spinal LTP (Table 2). PLC may induce Ca^2+ ^release from intracellular stores via IP3 receptors, providing part of the intracellular Ca^2+ ^rise necessary for LTP induction [4,17]. Ca^2+ ^release from intracellular stores via ryanodine receptors (RyRs) has also been shown to be necessary for spinal LTP induction [4,7,108].

Activation (phosphorylation) of mitogen-activated protein kinases (MAPKs) under different persistent pain conditions is involved in the induction and maintenance of pain hypersensitivity. In particular, nociceptive activity induces phosphorylation of spinal extracellular signal-regulated kinase (ERK) via multiple neurotransmitter receptors. Activated ERK, using different second messenger pathways, regulates the activity of glutamate receptors and potassium channels and induces gene transcription [109], and is therefore positioned to participate in both LTP induction and maintenance. Indeed, inhibition of ERK phosphorylation prevents LTP induction by HFS. This is likely to rely on neuronal ERK phosphorylation as HFS leads to a transient increase of phosphorylated ERK followed by a lasting increase of phosphorylated cAMP response element binding protein (CREB) in ipsilateral spinal dorsal horn neurons, but not in glial cells [110]. In contrast, block of c-Jun N-terminal kinase (JNK) and p38 MAPK does not prevent LTP induction [111].

Less is presently known about the intracellular signal transduction pathways required during induction of opioid-withdrawal LTP. While CaMKII does not seem to be necessary, block of PKC or RyRs has been shown to prevent LTP induction by opioid withdrawal [21].

#### Glia cells

Both microglia and astrocytes have a role in the generation and maintenance of hyperalgesia following inflammation or nerve injury [112]. Consistently, HFS or LFS of the sciatic nerve induce activation of spinal glia cells [15,113], and administration of an unspecific (fluorocitrate) or a microglia-specific glial metabolism inhibitor (minocycline) prevents induction of spinal LTP by HFS. At higher doses of these blockers, LTD is induced by HFS instead of LTP [113,114], suggesting that spinal glia have a role in the determination of the direction of synaptic plasticity. Similarly, the long-lasting facilitation of presynaptic excitation induced by LFS, as quantified by optical imaging, is prevented by glial metabolism inhibitors [32].

Microglia can be activated, e.g., by ATP that is released by primary afferent fibres, interneurons or astrocytes [115-117]. Activated microglia release proinflammatory cytokines, such as tumor necrosis factor α (TNF-α) and interleukin 6 (IL-6), which increase excitability of spinal neurons [118-121]. Spinal application of ATP induces LTP which depends on activation of microglia via P2X4 receptors and subsequent activation of p38 MAPK in microglia [122]. Similarly, bath application of the P2X receptor agonist αβmeATP leads to long-lasting facilitation of excitation in superficial dorsal horn (quantified by optical imaging) which is prevented by blocking glial metabolism or block of p38 MAPK or by administration of antibodies against the pro-inflammatory cytokines TNF-α and IL-6 [123].

Recent studies have shown that peripheral nerve injury induces activation of Src-family kinases (SFK) exclusively in spinal dorsal horn microglia [124]. Similarly to the effect of minocycline, blockers of SFKs not only prevent LTP induction following HFS, but instead lead to induction of LTD, an effect that is not present during simultaneous application of TNF-α [113]. Together, these results show that activation of microglia is necessary for the induction of HFS-induced LTP, and that stimulation of microglia by ATP is sufficient for the induction of spinal LTP. However, HFS-induced LTP and ATP-induced LTP seem to use different signal transduction pathways as ATP-induced LTP is blocked by p38 MAPK inhibitors while HFS-induced LTP is not [111,122]. In addition, spinal application of BDNF, which induces LTP of C-fibre evoked field potentials, activates microglia and up-regulates *p*- SFKs and *p*-p38 in microglia. Pre-treatment with minocycline, SFKs inhibitors or p38 MAPK inhibitors prevents both microglial activation and spinal LTP induced by BDNF [12].

Astrocytes are in close contact to neuronal synapses where they actively regulate synaptic transmission, e.g. by reuptake of glutamate from the synaptic cleft by the glutamate transporter 1 (GLT-1) [125-127]. Inhibition of GLT-1 prevents induction of spinal LTP following HFS [128]. This effect could be mimicked by intrathecal application of exogenous glutamate, suggesting that accumulation of glutamate in the synaptic cleft impairs LTP induction. Interestingly, this does not seem to be due to glutamate excitotoxicity [128]. It has been suggested that over-activation of NMDA receptors impairs LTP [129]. Indeed, impaired hippocampal LTP induction in GLT1-/- mice could be overcome in the presence of low doses of NMDA receptor antagonists [129]. Similarly, the induction of spinal LTP in the presence of fluorocitrate could be restored by application of low concentrations of an NMDA receptor antagonist [114].

### Interference with spinal LTP consolidation and modification of established spinal LTP in rodents

In the clinical context, patients often present with already established hyperalgesia, e.g. in the form of chronic pain. If LTP indeed contributes to certain forms of chronic pain, then the question arises how established LTP can be therapeutically modified. Reduction of synaptic strength during established LTP may be differentiated into transient ("symptomatic") and permanent ("causal") approaches. Symptomatic approaches will temporarily suppress synaptic transmission at the potentiated synapse but not affect the causal processes that maintain LTP, so that synaptic strength will return to elevated levels after wash-out of the drug. In contrast, causal approaches will reverse the intracellular modifications that maintain LTP and thus permanently revert (depotentiate) synaptic strength towards normal values.

In hippocampus, the maintenance of LTP induced by electrical stimulation can be divided into two distinct phases [107,130]. The early phase of LTP (E-LTP) sets in immediately after LTP induction but gradually fades away over the first few hours. It involves modification of pre-existing proteins like phosphorylation of synaptic AMPA receptors [131]. Consolidation of LTP requires expression of the late phase of LTP (L-LTP), which slowly develops during the hours after LTP induction and relies on de novo protein synthesis and gene transcription, e.g. resulting in the insertion of new AMPA receptors in the subsynaptic membrane [132]. According to the different mechanisms underlying the two phases of LTP, they may be affected by different drugs. In the rat spinal cord, the late, protein-synthesis-dependent consolidation phase of LTP slowly develops during the first few hours after stimulation, reaching its full expression between 3 and 6 hours after LTP induction [133]. Some drugs do not affect LTP induction but selectively interfere with spinal LTP consolidation by inhibiting the development of L-LTP when given before spinal LTP induction (antagonists at D1/D5 dopamine receptors, TrkB receptors, poly-ADRPTs, see Table 3). Other drugs induce a slow decay of LTP when given very early (15 min) but not later (30 min) after LTP induction (inhibitors of PKA, PKC, ERK, see Table 3). Kinetics and time course suggest that these drugs act by interfering with L-LTP development while leaving established E-LTP unaffected.

**Table 3:** Targets for interference with LTP consolidation and modification of established LTP.

								Start of drug application					
	Target	Substance	Action at target	HFS	LFS	*in vivo*	*in vitro*	Before LTP induction	During early phase (0-2 h)	During late phase (≥ 3 h)	Effect on L-LTP (unless stated otherwise)	Comments	References
NMDAR	NMDAR	MK 801, ketamine	antagonist	•		•			•		**0**		[101,134]

VGCC	α_2_δ-subunit	Gabapentin		•		•			•		**X/? (E-LTP/L-LTP)**		[65]

NK1R	NK1R	RP67580, 703,606	antagonist	•		•			•		**0**		[3]

GABA_A_R	GABA_A_R	3-APSA	agonist	•		•			•		**X/0** **(E-LTP/L-LTP)**		[70]
	GABA_A_R	Diazepam*, midazolam	Current amplifier	•		•			•	•	**X**	Depression not reversed by bicuculline	[70]

Opioid receptors	μ-opioid receptors	Morphine	agonist	•		•			•		**X/?** **(E-LTP/L-LTP)**		[65]

Descending inhibition	α_2_-adrenergic receptor	Clonidine	agonist	•		•			•	•	**X**	Biphasic depression	[90]
	D1/D5 dopamine receptor	SCH 23390	antagonist	•		•		•			**X**		[91]

Anaesthetic gases		Isoflurane		•	•	•		•			**0**	Drug present during entire experiment	[4,6,7]

NO-pathway	NOS	L-NAME	inhibitor	•		•			•		**0**	Deep dorsal horn	[29]
	extracellular NO	hemoglobin	scavenger	•		•			•		**0**		[29]
	sGC	ODQ	inhibitor	•		•			•		**0**		[29]
	mono-, poly- ADRPT	Benzamide	inhibitor	•		•		•			**X**		[29]

Adenosine receptors	A1 receptor	Cyclopentyladenosine	agonist	•		•			•		**X**	Superficial/deep dorsal horn. Drug inhibits LTP at both A-fibre and C-fibre synapses	[142]

Neurotrophins	TrkB receptor	K252a, TrkB- Fc	Trk inhibitor, BNDF scavenger	•	•	•		•			**0/X**	Blocks development of L-LTP in response to LFS but not HFS	[140]

EphR-ephrin signalling	EphB R	EphB1-Fc	antagonist	•		•			•		**0**		[102]
	EphB R	EphrinB1-Fc	agonist	•		•		•			**0**		[102]

Signal transduction pathways	CaMKII	KN-93, AIP, NK-62	inhibitor	•		•		•	•		**X/0**	Drugs inhibit LTP when administered at 60 min but not at 3 h after LTP induction	[143]
	PKA	Rp-CPT- cAMPS	inhibitor	•		•			•		**X/0**	Drugs inhibit LTP when administered 15 min but not 30 min after LTP induction	[143]
	PKC	Chelerythrine, Gö 6983	inhibitor	•		•			•		**X/0**		[143]
	MEK (ERK phosphorylation)	PD 98059	inhibitor	•		•			•		**X/0**		[110]
	Protein synthesis	Anisomycin, cycloheximide	inhibitor	•		•		•			**X**		[133]

Counterirritation		Prologed Aδ-fibre burst stimulation		•		•			•		**X/0** **(E-LTP/L-LTP)**		[144]
				•		•				•	**Potentiation**		[144]
		Repeated Aδ-fibre burst stimulation		•		•		•	•	•	**X/?** **(E-LTP/L-LTP)**	Cumulative depression	[36]

* Experiments that fulfilled the following criteria: (1) induction of LTP by HFS or LFS or natural noxious stimulation, (2) application of the drug during established late-phase LTP depresses LTP and (3) depression of LTP maintenance not terminated by application of an antagonistic drug (see text for explanation)

**X**, complete block or significant inhibition of LTP maintenance

**0**, no effect on LTP maintenance

Although the time course of the different phases of LTP in humans is currently unknown, modification of fully established L-LTP is presumably most important for possible clinical applications. Thus, animal experiments identifying drugs or interventions of possible clinical interest for the causal treatment of established LTP-associated hyperalgesia should be designed as follows: (1) induction of LTP by HFS, LFS, natural noxious stimulation or opioid withdrawal, (2) application of the drug during fully established L-LTP (i.e. at least 3 h, better 6 h after LTP induction [133]) and (3) if LTP is depressed, true reversal should be differentiated from prolonged drug action by application of an antagonistic drug to ensure that the effect persists after the drug action has been terminated. Alternatively, recording should be continued for a time period ensuring complete washout of the drug. Few studies have tested the effect of drugs or interventions during established L-LTP (≥ 3 h after LTP induction, see Table 3). Currently, only two drugs have been identified that depress established L-LTP (diazepam and clonidine), and only for diazepam, true reversal of L-LTP has been corroborated by use of an antagonistic drug.

**Figure 2:**
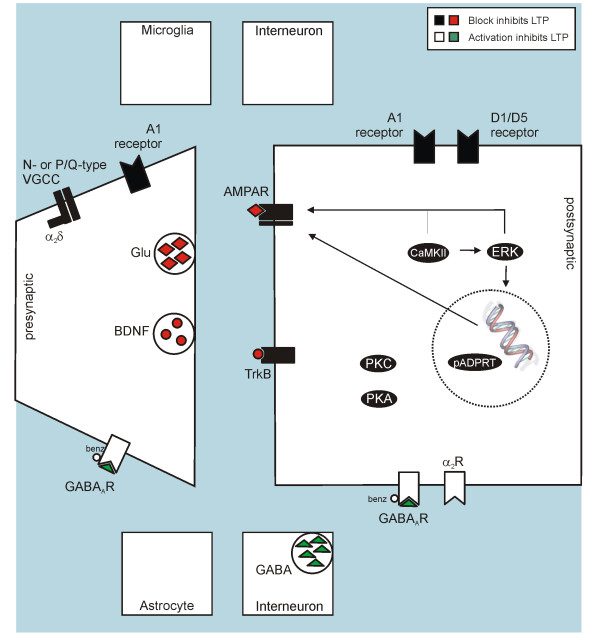
**Targets for modification of established spinal LTP in rodents**.

Targets for modification of LTP during the maintenance phase are summarized in Table 3, illustrated in Figure 2 and are discussed below.

#### Glutamate receptors

Despite the prominent role of the NMDA receptor in induction of most forms of spinal LTP, it does not seem to be required during the maintenance phase of LTP. Blockade of NMDA receptors with the non-competitive NMDA receptor antagonist MK-801 30 min after LTP induction in mice does not interfere with LTP maintenance [101]. Similarly, systemic pre-treatment with S(+)-ketamine effectively prevents LTP induction, but does not affect established LTP when given 60 min after conditioning stimulation [134].

#### VGCCs

The auxiliary VGCC subunit α_2_δ is a target of gabapentin and pregabalin, drugs successfully used in the therapy of neuropathic pain [59]. Their exact mechanisms of action remain unknown. Part of their action may be presynaptic, possibly affecting VGCCs of the N-type or P/Q-type on central terminals of C-fibres, as gabapentin reduces the release of substance P and CGRP from rat spinal cord slices after inflammation [64]. In addition, systemic gabapentin has been shown to activate descending noradrenergic systems, inducing spinal noradrenaline release [63,135] that has the potential to reduce synaptic transmission at nociceptive spinal synapses both at presynaptic and postsynaptic sites [136]. Evidence is converging that gabapentin has little effect on basal synaptic transmission or acute pain but inhibits established neuropathic or inflammatory pain [60-64]. Consistently, gabapentin depresses established LTP (given 60 min after LTP induction) but does not affect LTP induction [65]. As this study used systemic application of gabapentin, it is not possible to decide if the observed effect was mediated by local action the spinal cord level or by modulation of descending pathways.

#### NK1 receptors

Block of NK1 receptors does not affect established LTP [3]. Consistently, block of NK1 receptors does not affect established hyperalgesia [69]. This is in line with the notion that substance P is released from primary afferents during repetitive stimulation such as HFS, but not at the low frequencies used for test stimulation (e.g., 0.1 Hz) [66,137].

#### GABA_A _receptors

While administration of a GABA_A _receptor agonist only evokes a transient depression of LTP, benzodiazepines applied early (30 min) or late (3 h) after LTP induction completely or partially reverse LTP. This seems to be due to true reversal rather than prolonged drug action, as the depression persists after application of antagonists at the benzodiazepine or GABA binding site of the GABA_A _receptor [70]. It has been proposed that reversal of LTP by benzodiazepines might be due to inhibition of the cAMP/PKA and/or the NO pathways [70].

#### Opioid receptors

Morphine, a non-selective μ-opioid receptor agonist with affinity to δ- and κ-opioid receptors as well [138] given intravenously leads to a strong and dose-dependent reduction of C-fibre-evoked field potentials 60 min after induction of LTP by HFS [65]. As μ-opioid receptor agonists also depress baseline synaptic transmission of C-fibre-evoked postsynaptic potentials [82], it is not clear if LTP is reversed by morphine or if responses are acutely depressed similarly to control responses.

#### Receptor systems targeted by descending pathways: Adrenergic and dopaminergic receptors

Clonidine, applied at a dose that does not affect basal synaptic transmission, partially depresses both developing and established L-LTP (tested 30 min and 3 h after LTP induction, respectively). This action is mediated by activation of α_2_-adrenergic receptors [90]. The depression is biphasic, with a fast phase lasting 3.5 hours and a slow phase lasting till the end of the experiments at up to 5 hours, and seems to be partially mediated by activation of cholinergic interneurons and the NO-pathway. It was not tested if depression was due to prolonged drug action or to long-lasting modification of intracellular processes.

Block of dopamine receptors of the D1/D5 subtype before spinal LTP induction selectively depresses L-LTP development, while activation of these receptors induces a slowly rising LTP that presumably corresponds to the L-LTP induced by electrical stimulation [91].

#### Neurotrophins

BDNF is constitutively synthesized in a subpopulation of unmyelinated primary afferents [93] and is released into the superficial layers of the spinal dorsal horn along with substance P and glutamate in an activity-dependent manner [94]. Among other actions, BDNF increases protein synthesis both globally and locally [139] and is therefore positioned to contribute to L-LTP. Indeed, inhibition of the action of BDNF before LTP induction selectively reduces the L-LTP (but not E-LTP) induced by LFS [140]. In addition, upregulation of BDNF in DRG neurons seems to be a prerequisite for the consolidation of nerve injury-induced LTP, probably involving a BDNF action on microglia [12].

#### Ephrins

Although intrathecal application of EphB-receptor antagonists inhibits the maintenance of thermal and mechanical hyperalgesia following inflammation or nerve injury, it does not affect maintenance of spinal LTP when applied 30 min after LTP induction [102].

#### NO pathway

In contrast to LTP induction, LTP maintenance is not dependent on NO production, NO diffusion through the extracellular space or sGC action. However, spinal application of an inhibitor of poly-ADPRTs before HFS stimulation interferes with LTP consolidation, preventing L-LTP development [29]. Poly-ADPRTs are primarily nuclear enzymes that attach multiple ADPribose moieties to their substrates. They have been associated with DNA repair but also with DNA transcription [141], possibly explaining their involvement in L-LTP.

#### Adenosine receptors

Recently, it has been shown [142] that block of spinal adenosine receptor 1 (AR1) by cyclopentyladenosine (CPA) completely depresses spinal LTP at C-fiber synapses when applied 60 min after HFS. As CPA also strongly depresses baseline C-fibre evoked responses, it is not clear if LTP is reversed or if responses are acutely depressed similarly to control responses.

The same study reports that HFS at C-fibre intensity also induces LTP at spinal Aβ-fibre synapses. Aβ-fibre LTP is depressed by CPA applied 60 min after HFS. As basal Aβ-fibre responses are only marginally depressed by CPA, this seems to be due to a specific action of CPA on the potentiated Aβ-fibre response. Further characterization of the origin of the Aβ-fibre evoked field potential (e.g. nociceptive or non-nociceptive spinal neurons) will be necessary before evaluating any role of Aβ-fibre LTP as a potential mechanism underlying hyperaesthesia or allodynia.

#### Intracellular signal transduction pathways

Inhibition of PKA, PKC or ERK phosphorylation induces a slow decay of spinal LTP when administered during the first 15 min after induction but not when administered at 30 min [110,143]. Kinetics and time course suggest that these drugs interfere with L-LTP development. Inhibition of CaMKII still led to a slow decay of LTP when administered at 60 min after LTP induction [143], suggesting that L-LTP development can also be prevented at this later time point. However, inhibition of CaMKII does not reverse established L-LTP at 3 h after LTP induction.

Signal transduction pathways have also been investigated in models of pharmacologically induced LTP that may selectively mimic the L-LTP component of HFS-/LFS-induced LTP. Because of their similarity to L-LTP, results are presented here rather than in the LTP induction section. Spinal application of BDNF selectively induces a slowly rising, protein-synthesis-dependent LTP that shares features with L-LTP induced by electrical stimulation [140]. However, the pharmacology of the two forms of LTP only partially overlaps. Both BDNF-induced LTP and HFS-induced LTP are prevented by ERK inhibitors and not affected by JNK inhibitors [111,140]. However, inhibiting p38 MAPK prevents BDNF-induced LTP but not HFS-induced LTP, also not at time points after LTP induction where an action on L-LTP should be clearly evident [111,140]. Similarly, application of TNF-α induces a slowly rising LTP in the spinal cords of neuropathic, but not normal animals. Development of this LTP is prevented by inhibition of NF-κB, p38 MAPK and JNK.

#### Counterirritation

LTP and LTD have been suggested to partially rely on opposite cellular mechanisms, e.g. the phosphorylation vs. dephosphorylation of target proteins like CaMKII [41]. Therefore, manipulations that induce LTD at spinal nociceptive synapses may be able to reverse established E-LTP (but not necessarily L-LTP) by reversing LTP-related phosphorylation. Indeed, prolonged burst stimulation of primary afferent Aδ-fibres, that induces LTD of C-fibre evoked field potentials, partially depotentiates LTP induced by HFS of primary afferent C-fibres when applied early (≤1 h) after LTP induction [36,144]. However, a single prolonged Aδ-fibre burst stimulation does not seem to interfere with the development of L-LTP, as the depotentiation lasts for less than two hours [144]. A single prolonged Aδ-fibre burst stimulation also does not reverse established L-LTP, but rather induces additional potentiation when given late (3 h) after LTP induction [144]. The magnitude of depotentiation cumulates over repeated sessions of Aδ-fibre stimulation, but is it not clear if L-LTP is affected under these conditions [36]. Stimulation of non-nociceptive Aβ-fibres induces neither LTD nor depotentiation of LTP [36].

## Translational aspects of LTP in nociceptive pathways

### Spinal LTP induced by noxious stimulation

In rodents, LTP in nociceptive spinal pathways can be induced by noxious stimulation. This has led to the notion that human pain following intense noxious stimulation, e.g. acute postoperative pain or chronic pain developing after an initial strongly painful event, may in part be due to LTP in spinal nociceptive pathways.

Clinical pain manifests as a variable combination of spontaneous pain, hyperalgesia and allodynia (Footnote: according to the new definition of allodynia proposed by the IASP task force in 2008, only pain induced by stimuli not capable of activating nociceptors is classified as allodynia. At present, brush-induced allodynia, that has been shown to rely on transmission via primary afferent Aβ-fibres [145], is the only established example of allodynia according to this new definition). In humans, intense noxious stimulation or tissue injury typically evoke thermal and mechanical hyperalgesia within the stimulated/injured region (primary hyperalgesia) and mechanical hyperalgesia and brush-induced allodynia within a larger surrounding region of non-injured skin (secondary hyperalgesia). While primary hyperalgesia reflects sensitization of nociceptive primary afferents and also includes central mechanisms, secondary hyperalgesia is thought to selectively rely on central (spinal and/or supraspinal) mechanisms [2,146]. In chronic pain, spread of hyperalgesia to sites distant from the initial site of injury or even affecting the whole body, manifesting as a general elevation of pain sensitivity, may occur [26,147-150].

Before discussing the possible implications of injury-induced LTP for human experimental and clinical pain, it is important to determine which of the above manifestations of pain may be due to or enhanced by spinal LTP. LTP at synapses between nociceptive primary afferent C-fibres and superficial spinal dorsal horn neurons amplifies nociceptive signals. Therefore, LTP can account for hyperalgesia and possibly for small reductions in nociceptive thresholds and increases in size of hyperalgesic area. Hyperalgesia to pinprick stimuli is a frequent finding in human experimental or clinical hyperalgesia. Under normal conditions, pinprick stimuli are thought to be conducted by Aδ-fibres [151]. It is presently not known if spinal LTP also affects Aδ-fibre mediated synaptic transmission. However, recent work shows that pinprick hyperalgesia after inflammatory or nerve injury can be mediated by a subclass of C-fibres [152], suggesting that pinprick hyperalgesia might also relay on spinal LTP at C-fibre synapses. Brush-induced allodynia is thought to rely on input via primary afferent non-nociceptive Aβ-fibres [145]. Whether maintenance or modulation of allodynia outside the stimulated or damaged area is dependent on C-fibre sensitization remains controversial [153-155]. Therefore, the LTP at spinal C-fibre synapses described in the present review is unlikely to solely account for the origin of brush allodynia, although it might contribute to its modulation or maintenance. Although LTP at C-fibre synapses cannot induce spontaneous pain, it may exacerbate spontaneous pain in the region of an injury. Spontaneous pain appears as the result of spontaneous activity in primary nociceptive afferents or central nociceptive neurons. Spontaneous activity in primary afferents, e.g. resulting from peripheral sensitization or from ectopic activity [1], may be amplified in the spinal cord if LTP is present, leading to enhanced pain intensity.

LTP has a homosynaptic component, expressed at the same synapse that was activated by the conditioning stimulation. Homosynaptic spinal LTP may contribute to primary, but not to secondary hyperalgesia. However, synaptic plasticity may in addition be heterosynaptic, i.e. spread to neighboring synapses that have not been directly affected by the conditioning stimulation [156-158]. Studies investigating spinal LTP in rodents typically use supramaximal stimulation of the whole nerve trunk (sciatic nerve *in vivo*, dorsal root *in vitro*), presumably activating all intact fibres and consequently reaching all functional synapses between these fibres and second order neurons. Therefore, it is currently not possible to conclude whether this type of LTP is purely homosynaptic or also includes heterosynaptic components.

However, there is some direct evidence that heterosynaptic LTP occurs in spinal cord. When descending inhibition is removed, conditioning stimulation of Aδ-fibres induces LTP of C-fibre-evoked field potentials [36]. In addition, HFS of the tibial nerve or injury of the gastrocnemius/soleus motor nerve induces LTP of spinal field potentials evoked by stimulation of C-fibres in the sural nerve [12].

Heterosynaptic LTP may rely on various mechanisms. One possibility is that increased intracellular Ca^2+ ^and second messengers spread intracellularly to neighboring nociceptive synapses within the same neuron and induce LTP at these synapses. In addition, several neuromodulators, e.g. ATP and BDNF, have been shown to induce LTP in the absence of conditioning stimulation of the input pathway [12,122]. Intense noxious stimulation is known to release BDNF and ATP into the spinal cord [94,159]. Diffusion of these substances through the extracellular space may induce heterosynaptic LTP at synapses and neurons not directly activated by the injury or conditioning stimulation and thus contribute to secondary hyperalgesia. In fact, heterotopic LTP has been shown to rely on release of BDNF in spinal cord [12]. It is not known how far these substances can diffuse through the spinal cord. At least, diffusion within the same segment to affect synapses in the termination territory of a neighbouring nerve is possible in rodents [12]. In contrast, diffusion within the spinal cord tissue to distant segments or affecting synaptic transmission in the entire spinal cord seems improbable. On the other hand, more widespread effects could result if sufficient concentrations of these substances reached the cerebrospinal fluid. Whether LTP induced by an initial painful event can account for the spread of hyperalgesia to distant sites of the body or for the generalized hyperalgesia typical for chronic pain [147,149,160-163] is presently not known. Therefore, this manifestation of clinical pain will not be discussed in the present paper.

### Spinal LTP induced by opioid withdrawal

It has recently been discovered that in rodents, LTP in nociceptive spinal pathways can also be induced by abrupt withdrawal from opioids [21]. It has therefore been hypothesized that LTP may also contribute to the clinically important phenomenon of hyperalgesia following opioid withdrawal [21,22,42]. Although this has not been demonstrated directly, opioid-withdrawal LTP would be expected to affect nociceptive synapses throughout all spinal segments. Although it seems likely that opioid-withdrawal LTP can also lead to exacerbation of preexisting hyperalgesia or spontaneous pain, this has not been directly studied so far.

### Section conclusions

In conclusion, spinal LTP induced by an initial injury or noxious input may contribute to both primary and secondary hyperalgesia. LTP may also contribute to exacerbation of spontaneous pain. However, LTP induced by an initial painful event cannot explain brush allodynia. LTP induced by abrupt opioid withdrawal is proposed to lead to generalized hyperalgesia, possibly also including exacerbation of preexisting hyperalgesia.

It must be emphasized that although the above described sensory phenomena are compatible with spinal LTP, they may also be explained by other mechanisms. This is especially the case in primary hyperalgesia, where a substantial part of the hyperalgesia has been demonstrated to rely on sensitization of primary afferents [146]. The presence of secondary hyperalgesia is not in itself proof of the existence of LTP (i.e. altered synaptic strength), as secondary hyperalgesia can - and has - also been explained by changes in neuronal excitability (e.g. changes in neuronal membrane excitability) as well as changes in segmental or descendng inhibitory control [1,2,27,88,112,164-167]. Definitive proof of the existence of LTP depends on the direct measurement of synaptic strength, which is currently not feasible in humans. Therefore, we will, for the time being, have to accept that evidence for the existence of LTP in human pain pathways will remain indirect and circumstantial.

The following sections contain a more detailed description of those manifestations of human clinical and experimental pain that may principally be due to or exacerbated by spinal LTP, and compares their pharmacology to the known pharmacology of LTP in rodents. As primary hyperalgesia is in most cases accompanied by sensitization of nociceptive nerve endings, we will focus on secondary hyperalgesia (i.e. mechanical hyperalgesia in unstimulated or undamaged tissues) because this, at least, can safely be assumed to be due to central mechanisms [146,168]. In order to provide relevance to the clinical situation, we will also mention the impact of secondary hyperalgesia induction - or its modulation - on clinical pain measures. Typical measures of clinical pain outcome are pain scores, particularly on movement, and analgesia consumption, particularly in the acute or postoperative context. However, it must be emphasized that such clinical measures reflecting subjective pain experience are regularly found to be only weakly correlated to alterations in pain processing as quantified by various forms of formal sensory testing [161,162,169,170].

## Human volunteer and clinical models of hyperalgesia and LTP in nociceptive pathways

### Human volunteer models (Table 4)

**Table 4:** Methods of experimentally inducing secondary hyperalgesia possibly involving LTP in human volunteers

Type of stimulation		Protocol	Comments	References
Electrical nerve stimulation: C-fibres	HFS	100 Hz for 1 sec (pulse width, 2 ms), repeated five times at 10 sec intervals	Stimulation protocol and stimulated fibre type equivalent to rodent HFS paradigms inducing LTP	[19,25,173-175]
	Ongoing IFS	intracutaneous continuous electrical skin stimulation at 5 HZ (pulse width, 0.5 ms)		[176,188,189,227,231,232,254,259,261,262]

				

Natural noxious stimulation		Skin incision	without local anaesthetic	[178]
		Chemical injury	e.g. capsaicin, formalin; with or without thermal rekindling	For review[146,168]
		Thermal injury	e.g. heat burn, sunburn/UV	For review [179,180]

Pharmacological stimulation		Opioid withdrawal (remifentanil) during ongoing IFS or after capsaicin injection	increase in hyperalgesia and allodynia induced by transdermal electrical stimulation or intradermal capsaicin injection on stopping opioid infusion	[188-190]
		Opioid withdrawal (morphine and hydromorphone)	acute opioid withdrawal (naloxone) in volunteers made tolerant to opioids	[187]

HFS, high frequency stimulation

IFS, intermediate frequency stimulation

#### Electrical HFS, a specific human volunteer model of stimulus-induced LTP

Based on the observation that HFS of C-fibres is able to induce spinal LTP in *in vitro *and *in vivo *animal models [3,171], Klein et al. applied similar patterns of electrical C-fibre HFS transcutaneously via a special punctuate ring electrode in human volunteers [19]. Using psychophysical testing, they were able to demonstrate primary and secondary hyperalgesia. The homotopic perceptual correlate was hyperalgesia to electrical stimulation of C-fibres in the conditioned area up to at least three hours after the end of conditioning stimulation, while the heterotopic perceptual correlates consisted of hyperalgesia to pin-prick stimulation (presumably mediated by Aδ- and/or C-fibres [151,152]) and allodynia to brushing (presumably mediated by Aβ-fibres [145]), both in the area adjacent to conditioning stimulation, and again lasting at least three hours.

These results have been confirmed and expanded in subsequent psychophysical studies by this group [25,172-174] which are summarised in Table 5. These studies further demonstrated that HFS produces a leftward shift in the stimulus-response curve for heterotopic pinprick stimulation, that higher HFS intensities result in greater mechanical pinprick hyperalgesia, and that the duration of heterosynaptic pinprick hyperalgesia has a mean half-life of 3.3 hours and disappears after a mean of 25.4 hours. A study by another group has recently confirmed these results, further demonstrating that HFS-induced changes in heterotopic processing are also reflected in altered evoked somatosensory potentials, including N1-P2 peak-to-peak and P300 amplitudes [175].

**Table 5:** Effects of C-fibre HFS on pain perception inside and outside the conditioned skin area

	Conditioned area (primary hyperalgesia)	Unconditioned area (secondary hyperalgesia)
**Thermal hyperalgesia**	no	no
**Electrical hyperalgesia**	yes	yes
**Pinprick hyperalgesia**	yes	yes
**Blunt pressure hyperalgesia**	yes	yes
**Brush allodynia**	yes	yes

Thermal hyperalgesia at the stimulation/injury site is a typical feature of primary hyperalgesia and has been demonstrated to be largely due to sensitization of primary afferents [146]. The complete absence of thermal hyperalgesia within the conditioned area in the human HFS model therefore suggests that the HFS paradigm does not produce appreciable peripheral sensitisation. The quantitative sensory testing profiles showed hyperalgesia to the same types of stimuli inside and outside the HFS-conditioned areas, with the magnitude of hyperalgesia being 30% less in the surrounding area than in the stimulated area, but with highly correlated magnitudes between both areas. These results suggest that homo- as well as heterotopic amplification of central (e.g. spinal) nociceptive responses results from the HFS stimulation paradigm in intact human subjects, compatible with spinal LTP induction [174]. The time course of the sensory phenomena demonstrated in the human HFS model corresponds to that of early LTP in animal models involving mainly post-translational mechanisms [25].

#### Other human volunteer models of stimulus-induced secondary hyperalgesia

Ongoing transdermal electrical stimulation at a high current density (5 Hz, 50 mA) to recruit "sleeping" mechano-insensitive class C-nociceptors has also been used to induce large and stable areas of pinprick secondary hyperalgesia and ongoing pain in human volunteers [176]. These "sleeping" nociceptors are considered to be the same ones involved in capsaicin-induced pain and secondary hyperalgesia [177]. The ongoing pain and secondary hyperalgesia caused by their recruitment has been shown to be stable for at least two hours, making this model particularly suited to the study of secondary hyperalgesia and its therapeutic manipulation.

Using a model of skin incision in human volunteers and pre/post-traumatic local anaesthetic injections, Kawamata et al. showed that the peri-incisional hyperalgesia to punctuate mechanical stimuli, once developed, is more or less independent of peripheral nociceptive input from the incision, demonstrating the central (e.g. spinal) origin of this form of hyperalgesia [178]. The time course of the secondary hyperalgesia in this model is comparable to that resulting from cutaneous HFS, reaching its maximum 30-60 min after incision and lasting at least 6 hours before returning to baseline [25,178]. Similar findings have been reported for thermal and for chemical injury (e.g. by capsaicin or formalin) (for review see [146,168,179,180]). In all these cases, areas of secondary punctuate mechanical hyperalgesia are present surrounding stimulated/damaged tissue, with characteristics consistent with spinal sensitisation, e.g. due to LTP induction.

#### Opioid-induced hyperalgesia in human volunteers

Abrupt withdrawal of opioids has recently been shown to induce spinal LTP in an *in vivo *rat model [21]. Such LTP could be expected to manifest as generalised hyperalgesia or possibly also as increases in pre-existing secondary hyperalgesia. That opioids can paradoxically induce hyperalgesia under a variety of circumstances, including precipitate withdrawal, has been increasingly recognised in animal studies over the last decade [181-186]. This phenomenon has now also been documented in human volunteer models, either by demonstrating generalised hyperalgesia (e.g. using the cold pressor task) after acute opioid withdrawal (via naloxone) in subjects previously made opioid-tolerant [187], or by demonstrating that acute withdrawal of an opioid (remifentanil) infusion increases the area of cutaneous secondary pinprick hyperalgesia previously induced by either electrical transdermal stimulation [188,189] or capsaicin injection [190].

### Human patient models

#### Stimulus-induced secondary hyperalgesia after surgery in patients

General anaesthesia without additional analgesia is not sufficient to protect the spinal cord intraoperatively from the strong noxious input accompanying surgery [6,7]. Thus such general anaesthesia will not prevent the induction of LTP in the spinal nociceptive pathways, a process likely to increase acute postoperative pain. Consistently, secondary hyperalgesia has been demonstrated to be present peri-incisionally in human patients after surgery using a variety of psychophysical testing techniques. Thus punctuate secondary hyperalgesia has been demonstrated after a variety of surgical procedures by a number of groups [160,161,191,192], who have demonstrated this hyperalgesia to persist at least 7 days after surgery. Other groups have confirmed the presence of such secondary peri-incisional hyperalgesia using either electrical stimulation or pressure algometry with a similar time course [163,169,193,194].

#### Stimulus-induced secondary hyperalgesia in chronic pain patients

The development of chronic pain after human surgery is associated with the persistence and spread of secondary hyperalgesia, as now demonstrated by a number of human clinical studies [160,161,163,192]. While LTP can be postulated to at least partially explain the persistent secondary hyperalgesia in this context, it presently does not explain the delayed spreading, generalizing hyperalgesia, as discussed above.

Hyperalgesia to mechanical and electrical psychophysical testing is also a feature of a wide variety of established chronic pain conditions, including low back pain [150,195], fibromyalgia [196-199], rheumatoid arthritis [200], osteoarthritis [201], chronic widespread pain [202], irritable bowel syndrome [203-205], pancreatitis [148,206,207], gallstones [208] and headache [209]. Again, both secondary and spreading hyperalgesia are found in this context, with LTP being a possible underlying mechanism for secondary hyperalgesia, but with a presently unknown role in spreading hyperalgesia. It should be emphasized that differentiating secondary hyperalgesia from spreading hyperalgesia is frequently difficult in chronic pain patients.

Many chronic pain patients have an element of neuropathic pain due to peripheral nerve damage [210]. Nerve damage will amplify nociceptive input as well as providing spontaneous nociceptive input, with the resultant intense and ongoing nociceptive barrage to the spinal cord being similar to LTP-inducing conditioning stimulation [211]. Thus the hyperalgesia associated with nerve damage in chronic pain patients may partially reflect LTP in spinal nociceptive pathways.

#### Opioid-induced hyperalgesia in patients

The phenomenon of opioid-induced hyperalgesia is increasingly recognised in patients (for review see [22]). Thus Joly et al. demonstrated larger postoperative areas of secondary peri-incisional hyperalgesia in patients undergoing major abdominal surgery receiving high-dose remifentanil infusion intraoperatively as compared to low-dose remifentanil [212]. It should be noted that these patients all received a loading dose of morphine before end of surgery (and thus before end of remifentanil infusion), followed by further postoperative morphine titration for pain, making the situation not exactly comparable with the opioid withdrawal model for LTP in rodents [21]. The described increases in hyperalgesia were accompanied by poorer postoperative analgesic response to opioids, a finding supported in other studies of intraoperative opioid supplementation [22]. Generalised reductions in pain thresholds and tolerance have further been documented in drug addicts on methadone maintenance [213] and even in chronic low back pain patients after one month on opioid treatment [214]. In rodents, spinal LTP has been demonstrated upon opioid withdrawal [21]. It is presently not known if prolonged exposition to opioids also induces LTP in spinal nociceptive pathways. In addition, other mechanisms such as reduced descending inhibition or enhanced descending facilitation also likely play a role for opioid-associated hyperalgesia [215-217].

## Pharmacology of human hyperalgesia: Prevention of human hyperalgesia induction

In animal models, a variety of interventions have been found to prevent LTP induction. These can be divided into four basic categories, discussed in detail above, namely interventions: 1) reducing basal synaptic transmission at the first nociceptive synapse; 2) directly interfering with NMDA receptor activation; 3) interfering with additional sources of activity-dependent intracellular Ca^2+ ^rise, and 4) interfering with intracellular pathways downstream from Ca^2+ ^influx. Predominantly interventions in the first three categories have been investigated in humans, this restriction is mainly due to the limited availability of appropriate substances approved for human use.

Conclusions about possible interference with LTP induction can most convincingly be drawn from studies of secondary hyperalgesia in the context of human volunteer studies, e.g. cutaneous electrical high frequency stimulation. Some information may also be obtained from effects on early postoperative hyperalgesia in patients. However, it should be noted that in most cases the duration of the surgical intervention means that the period under investigation will also include the early phase of LTP - and may even include the later consolidation phase of LTP. Thus for the purposes of this review, discussion of prevention of LTP induction in the clinical human context will of necessity include the consolidation phase of LTP. A summary of interventions interfering with LTP induction (and possibly early consolidation phase) is provided in Table 6.

**Table 6:** Targets for prevention of secondary hyperalgesia induction in humans.

Target	Substance	**Action at target**	**Volunteer model effect**	Volunteer model used	**Clinical effect**	Clinical context	References volunteers	References clinical
**Stimulus-induced hyperalgesia**								

**Opioid receptors**	alfentanil	agonist	**X**	capsaicin	n/a	n/a	[218,219]	
	fentanyl	agonist	n/a	n/a	**X**	tested 5 days after back surgery	--	[169,193]
	remifentanil	agonist	**X**	heat/capsaicin	n/a	n/a	[222]	
	morphine	agonist	**X**	capsaicin heat/capsaicin burn	**X**	tested 24 h after hysterectomy	[220,221,224]	[233]
	hydromorphone	agonist	**X**	heat/capsaicin	n/a	n/a	[223]	
**Locoregional anaesthesia**	epidural anaesthesia	multiple	n/a	n/a	**X**	tested 3 days after colon surgery	--	[161]
	lidocaine	Na^+^-channel antagonist	**X**	skin incision capsaicin	n/a	n/a	[168,178]	
**Na^+ ^channels, systemic**	lamotrigine	antagonist	**0**	capsaicin	n/a	n/a	[225]	
	4030W92	antagonist	**0**	capsaicin	n/a	n/a	[225]	
**NK1R Descending inhibition**	aprepitant	antagonist	**0**	transdermal electric	n/a	n/a	[227]	
	desipramine	noradrenaline/5-HT reuptake inhibitor	**0**	capsaicin	n/a	n/a	[226]	

**NMDAR**	ketamine	antagonist	**X**	HFS/LTP capsaicin burn	**X**	tested 7 days after renal surgery, 3 days after colon surgery	[20,224,252]	[160,191]

**VGCC**	pregabalin	modulation at α_2_δ-subunit	**X**	transdermal electric	n/a	n/a	[227]	
	gabapentin	modulation at α_2_δ-subunit	**X/0**	transdermal electric, capsaicin	n/a	n/a	[228-230]	

**Opioid-induced hyperalgesia**								

**General anaesthesia**	propofol		**0**	transdermal electric/RW	n/a	n/a	[232]	
**α_2_-adrenergic receptors**	clonidine	agonist	**0**	transdermal electric/RW	n/a	n/a	[189]	
**COX-2**	parecoxib	antagonist	**0**	transdermal electric/RW	n/a	n/a	[231]	

**NMDAR**	ketamine	antagonist	**X**	transdermal electric/RW	**X**	tested 48 hours after major abdominal surgery with high-dose remifentanil infusion	[189]	[212]

**X**, complete block or significant inhibition of secondary hyperalgesia induction

**0**, no effect on secondary hyperalgesia induction

RW = remifentanil withdrawal

n/a = not available

### Human volunteer models

#### Prevention of stimulus-induced secondary hyperalgesia

##### Opioid receptor agonists

Application of systemic opioids is one of the classic approaches to achieve reduced synaptic transmission at the first nociceptive synapse, and has been demonstrated to prevent or reduce LTP induction in animal models (Table 2). Using a tailored infusion of alfentanil to produce plasma concentrations of 75 ng ml^-1 ^before capsaicin injection in a human volunteer model, Wallace et al. demonstrated reductions in capsaicin-induced stroking hyperalgesia (or allodynia) and in ongoing pain [218], with similar results being obtained for alfentanil by other researchers [219]. Similarly, Wang et al. showed that, in comparison to placebo, the area of secondary hyperalgesia is reduced by about 24% at 240 min post capsaicin by 10 mg of intravenous morphine applied 25 min prior to capsaicin [220]. Other authors have achieved comparable results for morphine [221], hydromorphone and remifentanil [222,223] using the heat/capsaicin sensitization model. Using the burn injury model, Warncke et al. also demonstrated significant reductions in secondary hyperalgesia using a morphine infusion started pre-lesionally [224]. It must, however, be said that it is difficult to distinguish between antihyperalgesia and analgesia in these circumstances.

##### Local anaesthesia and block of fast Na^+ ^channels

Another way of reducing nociceptive input is by local anaesthesia to the damaged tissues involved. In an incisional model in human volunteers, Kawamata et al. demonstrated that local anaesthesia administered before skin incision inhibited the development of secondary hyperalgesia, while post-incisional block did not [178]. Similar results have been found regarding the secondary hyperalgesia surrounding intradermal capsaicin injection [168]. However, systemic application of lamotrigine or 4030W92, thought to provide a use-dependent block of fast Na^+ ^channels, including those on peripheral nerve fibres, has not been shown to inhibit secondary hyperalgesia development when given prior to intradermal capsaicin [225].

##### NMDA receptor antagonists

LTP induction has been demonstrated to be dependent on NMDA receptor activation in animal models. The effects of ketamine, a non-specific NMDA receptor antagonist, have been studied in the previously presented specific human volunteer model of LTP induction via electrical cutaneous HFS [20]. The study demonstrated that low doses of ketamine (0.25 mg kg^-1^) given prior to HFS were able to prevent development of hyperalgesia to electrical stimulation within the HFS area, but not pinprick hyperalgesia or tactile allodynia in the area adjacent to HFS. The authors concluded that homotopic hyperalgesia due to HFS is sensitive to NMDA receptor blockade, and represents the human equivalent of the "classic" form of LTP seen in animal models. However, the heterotopic secondary hyperalgesia is not NMDA receptor sensitive, and may thus be the correlate of NMDA receptor independent forms of LTP and/or other central mechanisms of pain amplification. In this context, it should be noted that ketamine is a "dirty" drug, with additional interactions with non-NMDA, acetylcholine (nicotinic and muscarinic), serotonin and opioid-receptors, as well as Na^+^- and Ca^2+^-channels. However, at the low concentrations used by Klein et al. ketamine may be considered relatively selective for the NMDA receptor.

In contrast, ketamine has been shown to reduce secondary hyperalgesia in other human volunteer models. Ketamine has been shown to reduce the area of secondary mechanical hyperalgesia compared to placebo using both intradermal capsaicin and burn injury models [218,224,226]. Ongoing pain from the capsaicin injection was reduced, without effect on area of primary heat hyperalgesia [218].

##### Voltage-gated calcium channel (VGCC) modulators (gabapentinoids)

The gabapentinoids pregabalin and gabapentin bind to the α_2_δ-subunit of VGCCs, possibly interfering with presynaptic transmitter release and/or postsynaptic Ca^2+ ^rise. The effect of chronic oral administration of pregabalin has been tested in the already-mentioned model of secondary hyperalgesia induced by electrical transdermal stimulation [227]. Pregabalin, titrated to 2 × 150 mg per day and given orally for 6 days prior to induction of hyperalgesia, was demonstrated to significantly reduce area of hyperalgesia in comparison to placebo. Using the same model, Segerdahl found that gabapentin applied for 24 hours significantly reduced the area of hyperalgesia compared to placebo, without any reduction in spontaneous or evoked pain intensity [228]. In a study using intradermal capsaicin after 15 days' application of gabapentin, Gottrup et al. demonstrated reduction of allodynia areas - but only a trend for pinprick hyperalgesia areas -compared to placebo, without any reduction in ongoing or evoked pain intensity [229]. Applying gabapentin for 10 days prior to intradermal capsaicin, Wallace and Schultheis showed no effect on secondary hyperalgesia as compared to placebo [230].

##### Antidepressants

Tricyclic antidepressants may modulate nociceptive inputs to the spinal cord by enhancing the action of descending monaminergic inhibitory mechanisms. In a study involving desipramine, a tricyclic antidepressant agent, its chronic application was unable to reduce the induction of secondary hyperalgesia by intradermal capsaicin [226].

##### NK1 receptor antagonists

NK1 receptor antagonists have been shown to be effective inhibitors of LTP induction in animal models (Table 2). However, in human volunteers, oral application of aprepitant, an NK1 receptor antagonist, titrated to 320 mg per day for 6 days, proved unable to significantly reduce secondary hyperalgesia induced by electrical transdermal stimulation [227].

##### Prevention of opioid-induced hyperalgesia

The pharmacological modulation of hyperalgesia induced by opioid withdrawal, demonstrated to be associated with LTP induction in rodents [21], has been extensively studied in a human volunteer model involving secondary hyperalgesia induced by electrical transdermal stimulation in combination with remifentanil infusion withdrawal [188]. It must be emphasized that this human model is not fully comparable to the rodent LTP induction model. The human model uses an increase in pre-existent stimulus-induced secondary hyperalgesia as endpoint for the opioid effect, while in the rodent model, hyperalgesia is induced by opioid withdrawal alone.

##### NMDA receptor antagonists

Animal models have shown that NMDA receptor block prevents LTP induction by opioid withdrawal [21]. Congruently, a human volunteer study using electrical transdermal stimulation to produce secondary hyperalgesia has demonstrated that the addition of S-ketamine to remifentanil infusion prevents the expansion of stimulus-induced hyperalgesia on acute opioid withdrawal [189].

##### Others

In the human model under discussion, neither the co-infusion of the anaesthetic agent propofol, the central α_2_-adrenergic receptor agonist clonidine, or of the selective COX-2 inhibitor parecoxib, could be shown to significantly reduce the increased area of stimulus-induced hyperalgesia following abrupt remifentanil infusion withdrawal [189,231,232]. Although it did not reduce this hyperalgesia, co-administration of clonidine did reduce rebound of the ongoing pain scores due to conditioning electrical transdermal stimulation after cessation of remifentanil infusion [189]. Currently, no data are available in rodents for these pharmacological targets regarding opioid-withdrawal induction of hyperalgesia.

### Human patient models

Perioperative sensory testing of the secondary hyperalgesia surrounding surgical incision is an attractive way of studying the time course of central pain amplification - and hence potentially LTP - in the clinical context. However, as already mentioned, it should be realised that due to the length of surgery, the effects of perioperative therapeutic intervention will not only influence LTP induction, but also its consolidation.

#### Prevention of stimulus-induced hyperalgesia

##### Opioid receptor agonists

Opioids, shown to be effective in inhibiting LTP induction in animal models, and in reducing secondary hyperalgesia in human volunteer models, are also effective in reducing peri-incisional secondary hyperalgesia in clinical surgical patients. Thus fentanyl applied before surgical incision has been shown to reduce the degree of secondary hyperalgesia five days after back surgery vs. placebo [169,193], and morphine given before incision has been demonstrated to reduce peri-incisional hyperalgesia vs. morphine given at the end of abdominal surgery [233]. In contrast, in these and other pre-emptive analgesia studies involving opioids, clinically significant effects on postoperative pain scores and analgesia consumption have proven difficult to demonstrate and remain controversial [162]. In this context, it is also worth noting the differences in the use of opioids between these studies and those investigating opioid-induced hyperalgesia. The studies investigating opioid-induced hyperalgesia generally involve the use of a short-acting opioid (typically remifentanil), given as an infusion producing relatively high and constant plasma levels, which is then abruptly discontinued at the end of surgery. In contrast, the pre-emptive analgesia studies quoted [169,193,233] entail the application of a bolus of a long-acting opioid in moderate dosages, producing peak plasma concentration with surgical incision, and then gradually tapering off as surgery progresses to its completion.

##### Locoregional anaesthesia/analgesia

The better blockade of neuraxial sensory input provided by epidural anaesthesia as compared to systemic application would be expected to further reduce basal synaptic transmission at the first nociceptive synapse and thus to more effectively depress spinal mechanisms of central pain amplification, including LTP. Lavand'homme et al. demonstrated that for colon surgery, the groups receiving perioperative epidural anaesthesia (local anaesthetic + opioid + clonidine) vs. purely intravenous perioperative analgesia showed considerably less incisional secondary hyperalgesia up to three days post-operatively [161]. Interestingly, the epidural groups with less early postoperative secondary hyperalgesia also showed less persistent and chronic pain up to one year postoperatively [161]. Similarly, intrathecal clonidine administered before incision reduced secondary hyperalgesia vs. saline placebo up to three days after colon surgery, with reduced secondary hyperalgesia again being associated with less persistence of pain (chronic pain) up to six months postoperatively [192].

##### NMDA receptor antagonists

In animal and human volunteer models, NMDA receptor blockade prevents LTP induction. Application of the non-competitive NMDA receptor antagonist ketamine before surgical incision has been shown to reduce postoperative peri-incisional hyperalgesia after renal and colon surgery [160,191], thus supporting the hypothesis that also in the clinical context, NMDA receptor blockade inhibits LTP induction. One of these studies again demonstrated that the associated reduction of postoperative secondary hyperalgesia was linked to lower incidences of chronic pain later on [160]. The efficacy of ketamine in improving perioperative pain outcomes is supported by extensive literature [234-239].

There are few data on the effects of other NMDA receptor antagonists used perioperatively. Ilkjaer et al. studied the use of preoperative oral dextromethorphan, also a non-competitive NMDA receptor antagonist, on early and late postoperative hyperalgesia and pain [240]. They were unable to demonstrate differences vs. placebo regarding either hyperalgesia or pain posteroperatively, probably due to inadequate dosage.

##### VGCC modulators (gabapentinoids)

In rodents, acute application of gabapentin interferes with LTP maintenance but not LTP induction [65]. To date we have been unable to find studies directly documenting effects of these drugs on postoperative secondary hyperalgesia. There is however, a considerable literature available documenting the positive effects of perioperative administration of gabapentinoids on postoperative pain outcomes, particularly acute, but also more long-term [241-245].

#### Prevention of opioid-induced hyperalgesia

##### Opioid receptor agonists

The effect of pre-emptive opioids in preventing opioid-induced hyperalgesia has not been studied so far using formal sensory testing methods. However, the application of a long-acting opioid before the start of a remifentanil infusion has not been shown to improve postoperative pain outcomes [246,247]. It has not been determined whether administration of a long-acting opioid before abrupt withdrawal prevents opioid-withdrawal LTP in rodents, but this seems likely as tapered opioid withdrawal does not induce LTP [21].

##### NMDA receptor antagonists

Regarding opioid-induced hyperalgesia, Joly et al. demonstrated in a clinical study in patients undergoing major abdominal surgery that the large postoperative areas of secondary peri-incisional hyperalgesia associated with high-dose intraoperative remifentanil infusion could be significantly reduced by the concomitant use of small-dose ketamine [212]. These data are supported by studies documenting effects of ketamine on opioid-induced hyperalgesia using acute clinical postoperative pain outcomes [248,249].

### Section summary and conclusions

#### Prevention of stimulus-induced secondary hyperalgesia

In the human literature just reviewed, we present data that hyperalgesia induction in human volunteer models and patients and LTP induction in rodents share a similar pharmacology, supporting the hypothesis that LTP in spinal nociceptive pathways is a cellular mechanism of hyperalgesia.

More specifically, there is good evidence that opioid μ-receptor agonists and nerve blockade by local anaesthesia (both reducers of first nociceptive synapse transmission) and NMDA receptor blockade by ketamine effectively inhibit secondary hyperalgesia induction in both volunteers and patients, congruent with animal model results. Similarly, modulation of descending inhibition by spinal application of the α-adrenergic agent clonidine has been demonstrated to inhibit both hyperalgesia induction in patients and LTP induction in rodents.

The evidence for the ability of gabapentinoids (titrated over several days) to inhibit induction of secondary hyperalgesia in humans is inconclusive with both positive and negative effects reported in the literature. In rodents, no effect of acute application of gabapentin was found on LTP induction. Titration over several days has been used in human studies to enhance tolerability. It cannot be excluded that this protocol also enhances the antihyperalgesic effects of gabapentinoids. Similar titration protocols have not been tested in rodents so far.

NK1 receptor antagonists prevent LTP induction in rodents but have no effect on induction of secondary hyperalgesia in humans. However, these studies may be difficult to compare because of different drug application schedules (titration for several days in humans vs. acute spinal application in rodents).

The comparison of pharmacology between human hyperalgesia induction and rodent LTP induction is summarised in Table 8.

#### Prevention of opioid-induced hyperalgesia

In agreement with the animal literature, both human volunteer and patient models of opioid-induced hyperalgesia show prevention of hyperalgesia induction by effects of NMDA receptor blockade using ketamine. In the human volunteer model, neither general anaesthetics (propofol), α-adrenergic agonists (clonidine) nor COX inhibitors (parecoxib) are effective in preventing the induction of opioid-induced hyperalgesia.

## Pharmacology of human hyperalgesia: Modulation of established human hyperalgesia

As mentioned in the section on animal models, LTP induction occurs in two phases. The early phase, involving modification of pre-existing proteins, sets in immediately after induction and then dies away over the first few hours. LTP consolidation occurs in the late phase, based on de novo protein synthesis and gene transcription, and is complete 3 - 6 hours after LTP induction in animal models.

Both causal and symptomatic approaches to modification of established LTP are principally possible. Causal approaches reverse intracellular events maintaining LTP, while symptomatic approaches temporarily inhibit synaptic transmission at the potentiated synapse without affecting intracellular processes maintaining LTP. A major difference between the two approaches would thus be whether hyperalgesia reappears after drug wash-out. A summary of interventions modifying established hyperalgesia in humans is provided in Table 7.

**Table 7:** Targets for modulation of established secondary hyperalgesia in humans.

Target	Substance	Action at target	Volunteer model effect	Volunteer model used	Clinical effect (neurop athic pain)	Comment re. hyperalgesia	References volunteers	References clinical
**Stimulus-induced hyperalgesia**								

**Opioid receptors**	alfentanil	agonist	**X**	transdermal electric	**X**	only ongoing and evoked pain	[176]	[263]
	alfentanil	agonist	**X/0**	capsaicin	n/a	n/a	[250-252]	n/a
	remifentanil	agonist	**X**	transdermal electric	n/a	n/a	[189]	n/a
	morphine	agonist	**0**	burn	n/a	n/a	[253]	n/a
	buprenorphine	Partial agonist- antagonist	**XX**	transdermal electric	n/a	n/a	[254]	n/a
**COX**	paracetamol, parecoxib	COX/COX2 inhibition	**XX**	transdermal electric	n/a	n/a	[259]	n/a
	ibuprofen	COX inhibition	**0**	burn	n/a	n/a	[260]	n/a
**Na^+ ^channels, systemic**	lidocaine	antagonist	**XX**	transdermal electric	n/a	n/a	[176]	n/a
	lidocaine	antagonist	**X**	capsaicin	**X**	only evoked pain	[256]	[263]
	lamotrigine	antagonist	**0**	heat/capsaicin	n/a	n/a	[223]	n/a
**General anaesthesia**	propofol		**X**	transdermal electric	n/a		[262]	n/a
								
**Adenosine receptors**	adenosine	agonist	**X**	transdermal electric	n/a	n/a	[261]	n/a
**Descending** **inhibition**	venlafaxine	noradrenaline/5-HT reuptake inhibitor	n/a	n/a	**X**	evoked pain + area	n/a	[267]

**NMDAR**	ketamine	antagonist	**X**	burn	**X**	ongoing and evoked pain + area	[250,253,255,256]	[263,265]
	ketamine	antagonist	**X/0**	capsaicin	n/a	n/a	[250-252,256]	n/a
	S-ketamine	antagonist	**XX**	transdermal electric	n/a	n/a	[176]	n/a
	dextro-methorphan	antagonist	**X**	burn	n/a	n/a	[257]	n/a

**VGCC**	gabapentin	modulation at α_2_δ-subunit	**X**	heat/capsaicin	**X**	n/a	[258]	n./a

**X**, complete block or significant inhibition of established secondary hyperalgesia during drug effect

**XX**, complete block or significant inhibition of established secondary hyperalgesia outlasting drug effect

**0**, no effect on established secondary hyperalgesia

n/a = not available

### Human volunteer models

#### Interference with stimulus-induced secondary hyperalgesia

##### Opioid receptor agonists

Koppert et al. have investigated the effect of a number of clinically available compounds on pre-existent secondary hyperalgesia in the context of their model of ongoing transdermal high current density electrical stimulation. As hyperalgesia was induced only 30 min before drug application, this model might be comparable to drug application during early but not late phase LTP. Using this model, Koppert et al. demonstrated that pure μ-opioid receptor agonists such as alfentanil and remifentanil reduced hyperalgesia during the period of application [176,189]. The fact that hyperalgesia reappeared after opioid washout strongly suggests a purely symptomatic effect on hyperalgesia and possibly underlying LTP. Conflicting results have been obtained after intradermal capsaicin, with one group reporting transient antihyperalgesic effects with intravenous alfentanil infusion [250], and others no effects for bolus or infusion application of alfentanil [251,252]. Using an infusion of morphine at 10 μg kg^-1 ^min^-1 ^started 30 min after burn injury, Schulte et al. were unable to detect antihyperalgesic effects 45 and 75 min after start of infusion [253]. Interestingly, the use of buprenorphine, a partial μ-receptor agonist and κ- and δ-receptor antagonist, in the transdermal high current density electrical stimulation model leads to a long-lasting reversal of hyperalgesia outlasting the end of drug application by almost 150 min [254]. Whether this is due to causal effects or a long duration of action of buprenorphine cannot be ascertained from the study.

##### Local anaesthesia

Regarding local anaesthesia to the damaged tissue, Kawamata et al. demonstrated that local anaesthesia administered after skin incision in volunteers did not inhibit secondary hyperalgesia, in contrast to pre-incisional block, which did [178].

##### NMDA receptor antagonists

Using a skin burn model in human volunteers, Ilkjaer et al. studied ketamine (intravenous bolus of 0.15 or 0.3 mg kg^-1 ^followed by a 135 min infusion at 0.15 or 0.3 mg kg^-1 ^h^-1^), compared to placebo infusion, and applied 15 min after lesioning [255]. Ketamine reduced the area of established primary and secondary hyperalgesia in a dose-dependent manner during the period of infusion - but not thereafter. Ketamine further reduced heat-evoked pain responses within the area of primary hyperalgesia, but had no effect on heat-evoked pain responses in skin at sites distant from the burn. Analogous positive results for ketamine have been found based on intradermal capsaicin [250,256], with other such studies failing to demonstrate antihyperalgesic effects with bolus application (0.07 or 0.29 mg kg^-1^) or targeted infusion (150 ng ml^-1^) [251,252]. A further study based on burn injury to the skin again found reductions in hyperalgesia with ketamine infusion (0.9 μg kg^-1 ^min^-1 ^for 45 min) [253]. The use of S-ketamine infusion (increasing concentrations; slope = 30 ng ml^-1 ^min^-1 ^over 10 min + 10 min plateau) in the context of the transdermal electrical hyperalgesia model also demonstrated significant antihyperalgesic and analgesic properties [176]. However, in this study, the antihyperalgesic effects of S-ketamine outlasted infusion end for at least one hour. These differences in results may be due to the higher doses of ketamine used, differences between ketamine and S-ketamine, or differences in the nature of hyperalgesia produced by the different models. As hyperalgesia was generally induced less than one hour prior to ketamine infusion, all these models again only can be compared to actions on early LTP.

Dextromethorphan, a non-competitive NMDA receptor antagonist too, has also been shown to reduce the area of secondary hyperalgesia induced by a burn injury [257].

##### VGCC modulators (gabapentinoids)

A single dose of gabapentin given 90 min after induction of secondary hyperalgesia using the heat/capsaicin model significantly attenuated the area of hyperalgesia compared to placebo, and also significantly reduced the area of hyperalgesia when given directly after rekindling some 90 min after initial induction [258]. As hyperalgesia was induced approx. 90 min prior to gabapentin application, this model is again comparable only to the action on early LTP.

##### Cyclooxygenase (COX) antagonists

The use of the central COX isozyme inhibitors paracetamol and parecoxib also resulted in long-lasting inhibition (at least 150 min after drug infusion) of transdermal electrically induced hyperalgesia [259]. Again, the study design makes it impossible to decide whether this is due to long duration of drug action or causal effects, i.e. permanent reversal of the mechanisms underlying hyperalgesia. The non-selective COX inhibitor ibuprofen applied after burn injury did not, however, reduce secondary hyperalgesia [260].

##### Others

Other substances which may affect primary nociceptive synaptic transmission studied by the Koppert group include systemic application of adenosine [261], propofol [262] and the Na^+ ^channel blocker lidocaine [176]. For both adenosine and propofol, the significant antihyperalgesic and analgesic effect did not outlast infusion, suggesting symptomatic effects. Lidocaine infusion, however, resulted in significant antihyperalgesic effects which outlasted the infusion by about 50 min. Gottrup et al. found similar effects for lidocaine infusion on pre-existing hyperalgesia induced by intradermal capsaicin [256]. Oral lamotrigine, a use-dependent Na^+ ^channel antagonist, has, however not been shown to have antihyperalgesic effects following heat/capsaicin sensitisation [223].

### Human patient models

If LTP is involved in the maintenance of some forms of chronic pain, then therapeutic manoeuvres modifying established LTP in animal models could be expected to impact the hyperalgesia associated with established chronic pain in patients. As already discussed above, we will restrict this discussion to secondary hyperalgesia (i.e. surrounding the initial lesion). Not many human clinical studies using formal sensory testing have been performed in this context; most are small and have been carried out in the context of patients suffering from chronic pain associated with peripheral nerve injury. This is a relevant model as LTP has also been shown to play a role in nerve-injury related pain in rodent models [13].

#### Interference with stimulus-induced secondary hyperalgesia

##### Opioid receptor agonists

A small number of studies have looked at the effect of the opioid agonist alfentanil, applied as a short intravenous infusion in patients exhibiting chronic pain linked to peripheral nerve injury. In all of these studies, allodynia as well as mechanical secondary hyperalgesia were studied. It is worth noting here that while secondary mechanical hyperalgesia is compatible with LTP-like mechanisms, Aβ-fibre mediated allodynia is unlikely to involve LTP at C-fibre synapses as a mechanism. Leung et al. applied alfentanil as a target-controlled infusion (target: 25, 50 and 75 ng ml^-1^) to patients with chronic neuropathic pain and demonstrated dose-dependent decreases in ongoing and von Frey-hair evoked pain without a decrease in area of secondary hyperalgesia, concomitantly with reductions in brush-evoked pain and area of mechanical allodynia [263]. Using a similar design (alfentanil target plasma concentration approx. 44 ng ml^-1^), Jørum et al. found similar results for mechanical allodynia, but did not study effects on mechanical hyperalgesia [264]. Neither of these studies investigated whether antihyperalgesic effects outlasted the end of drug infusion.

##### NMDA receptor antagonists

To date, the only NMDA receptor antagonist studied for its effects on secondary hyperalgesia in the context of neuropathic pain is ketamine. Gottrup et al. also investigated ketamine (0.24 mg kg^-1 ^over 30 min), finding that it reduced ongoing pain as well as magnitude of secondary pinprick hyperalgesia and brush allodynia [265]. Using target-controlled infusions of ketamine (50-150 ng ml^-1^), Leung et al. demonstrated reductions in area of secondary pinprick hyperalgesia together with reduction in allodynic area and allodynia (i.e. brush-evoked pain) [263]. Two studies found comparable results for ketamine regarding mechanical allodynia, but did not study effects on mechanical hyperalgesia [264,266]. None of these studies reported effects outlasting the period of drug infusion.

##### Antidepressants

As already mentioned, antidepressants may also modulate spinal nociceptive input via descending monoaminergic mechanisms. In quite a large study (n = 55) Yucel et al. studied the effects of chronic venlafaxine administration (8 weeks) on secondary mechanical hyperalgesia in chronic neuropathic pain patients [267]. Compared to placebo, venlafaxine significantly decreased pin-prick hyperalgesia and its area; the same was the case for brush allodynia. The fact that both hyperalgesia and Aβ-fibre mediated allodynia were affected suggest that a significant proportion of the venlafaxine effects must be the results of mechanisms other than LTP at C-fibre synapses in this context.

##### Na^+ ^channel blockers

Gottrup et al. studied the effects of intravenous lidocaine (5 mg kg^-1 ^over 30 min) in neuropathic pain patients [265]. They were able to demonstrate that the Na^+ ^channel blocker lidocaine reduced evoked pain to repetitive pinprick stimuli, without effects on ongoing pain or brush-evoked pain (allodynia). The study did not investigate areas of hyperalgesia or allodynia.

### Section summary and conclusions

Comparison of the human and animal literature presented above shows that established rodent LTP and established human hyperalgesia share a similar pharmacology with one major exception.

μ-opioid agonists decrease established secondary hyperalgesia in human volunteer and patient models. Gabapentinoids, too, have been shown to be effective against established hyperalgesia in human volunteer models. This is consistent with the results from animal models where μ-opioids and gabapentinoids suppress LTP during LTP maintenance phase.

Antidepressants have been shown to be effective against established hyperalgesia in pain patients. As antidepressants and central α_2_-adrenergic-agonists such as clonidine share central monoaminergic mechanisms, the antihyperalgesic effectiveness of antidepressants in humans might find its animal equivalent in the effectiveness of clonidine in inhibiting established LTP.

However, in animal models, NMDA receptor blockade has no effects on established LTP, which contrasts with the evidence presented that NMDA receptor blockade by ketamine interferes with established secondary hyperalgesia in both human volunteer and patient models. One possible hypothesis explaining this difference would be that in the context of the human models presented, ongoing nociceptive input - albeit at a low level - leads to continuing induction of LTP, contributing to the maintenance of LTP, and thus explaining the sensitivity of apparently established secondary hyperalgesia to NMDA receptor blockade. Alternatively, LTP may be only one of various central mechanisms contributing to established human hyperalgesia and chronic pain, with alternative, NMDA receptor sensitive mechanisms participating in the maintenance phase.

COX inhibition, general anaesthetics, intravenous lidocaine or adenosine, have all been shown to be effective against established hyperalgesia in human volunteer or patient models but have not been tested in animal models of LTP.

Both in human and animal studies, it has often not been tested whether inhibition of established LTP/hyperalgesia outlasts drug effects, precluding differentiation between symptomatic (acute antinociceptive or antihyperalgesic) and causal (reversal of LTP/hyperalgesia) effects. In humans, there is some evidence that for ketamine, lidocaine, paracetamol/parecoxib and the atypical opioid buprenorphine, antihyperalgesia may outlast drug effects, suggesting that causal actions might be operating. However, all of these studies have been performed only in the ongoing transdermal electrical stimulation model, and their applicability to other models and the clinical context remains to be proved.

Our conclusions regarding LTP in rodents vs. humans and its pharmacological modulation are contrasted and summarised in Table 8.

**Table 8:** Comparison of the pharmacology of stimulus-induced rodent LTP and human secondary hyperalgesia or clinical pain

Target, action	Rodent LTP	Human models of secondary hyperalgesia	Human clinical pain		Comments
				
			QST: secondary hyperalgesia	Clinical response: pain report	
**Induction (Human postoperative pain)**					

μ-opioid receptor agonist	**X**	**X**	n.t. (area) **X **(thr/rating)	**controversial**	

NMDA receptor antagonist	**X**	**X^1^**	**X **(area) **X **(thr/rating)	**X**	Ketamine also blocks OIH induction in rodents and humans

α-adrenergic receptor antagonist	**X**	**X**	**X **(area) n.t. (thr/rating)	**X**	Acute spinal application of clonidine in humans

NK1 receptor antagonist	**X**	**0**	n.t.	n.t.	Acute spinal application in rodents vs. chronic oral application in humans

Modulation of α_2_δ VGCC subunit	**0**	**X/0**	n.t.	**X**	Acute spinal application in rodents vs. chronic oral application in humans

**Maintenance (Human chronic neuropathic pain)**					

μ-opioid receptor agonist	**X**	**X**	**0 **(area) **X **(thr/rating)	**X**	

NMDA receptor antagonist	**0**	**X**	**X **(area) **X **(thr/rating)	**X**	

Modulation of α_2_δ VGCC Subunit	**X**	**X**	n.t.	n.t.	

α-adrenergic receptor agonist/noradrenaline reuptake inhibitor	**X**	n.t.	**X **(area) **X **(thr/rating)	**X**	Clonidine (rodents) vs. venlafaxine (humans)

**X**, induction/established state blocked by action at target

**0**, induction/established state not blocked action at target

n.t., not tested

QST, quantitative sensory testing

area, area of secondary hyperalgesia mapped using QST

thr/rating, threshold or rating of evoked pain as determined by QST

OIH, opioid-induced hyperalgesia

^1^, including action on LTP of human pain perception

## Conclusions

In rodents, LTP of spinal nociceptive pathways is a cellular model of long-lasting (but not necessarily irreversible) hyperalgesia induced by noxious stimulation or opioid withdrawal. Both noxious stimulation and opioid withdrawal also induce prolonged pain amplification in the human experimental and clinical context. Noxious stimulation of a pattern that is LTP-inducing in rodents induces hyperalgesia in humans. Of the various manifestations of human experimental and clinical pain, some may be related to LTP while others cannot be explained by this mechanism. For prolonged pain after noxious stimulation, LTP may explain hyperalgesia and possibly exacerbation of spontaneous pain at or surrounding the initial lesion site, but not Aβ-fibre mediated allodynia (e.g. brush allodynia). For prolonged pain after opioid withdrawal, LTP may explain generalized hyperalgesia, possibly including exacerbation of preexisting hyperalgesia. Direct proof of the involvement of spinal LTP in pain conditions is at present not feasible in humans. However, the current review shows that rodent spinal LTP and human hyperalgesia share a similar pharmacology, further supporting the role of rodent spinal LTP as a model for prolonged pain and hyperalgesia in humans.

One major issue with respect to the role of spinal LTP as a model of persisting pain in humans is its unknown duration. In principle, LTP may last for hours, days, months or throughout the lifespan of an animal. So far, behavioural correlates of spinal LTP in rodents or human volunteers seem to be in the range of several days, compatible with, e.g., acute postoperative pain but not with chronic pain. One hypothesis would be that in chronic pain, LTP is prolonged by various factors that might "boost" the maintenance of LTP, counteracting its natural decline. Examples might include decreased activity of endogenous antinociceptive systems or the presence of ongoing intermittent low-level nociceptive input from the periphery. Investigating these possibilities in spinal LTP in rodents or LTP of pain perception in humans might be a fruitful approach for future studies.

Inhibition of the induction of hyperalgesia by noxious stimulation is important for prevention of both acute and chronic postoperative pain. Standard general anaesthesia alone is not sufficient to protect the spinal cord from the strong noxious input during surgery. Only locoregional anaesthesia techniques appear to offer some protection in this context. Several drugs have been identified that inhibit induction of rodent LTP and human hyperalgesia, e.g. NMDA receptor antagonists, μ-opioid receptor agonists and clonidine. From the rodent LTP literature, novel promising approaches may include antagonists at T-type VGCCs and (possibly subtype-selective) antagonists at GABA_A _receptors.

Identification of drugs that reverse the central processes contributing to chronic pain maintenance would be a major advance in chronic pain treatment. Assuming that spinal LTP contributes to chronic pain, animal experiments will likely contribute to the identification of such drugs by investigating their action on established LTP. Differentiating acute antihyperalgesia ("symptomatic" drug action) from long-lasting reversal of the mechanisms maintaining hyperalgesia ("causal" drug action) requires extending the observation period beyond the time of drug action termination by washout or application of an antagonist. In addition, investigation of drug actions on late phase LTP (> 3 h after LTP induction) is likely of greater clinical relevance than on early phase LTP. Up to now, among the compounds that are also in clinical use, rodent studies have identified two drugs which suppress late-phase LTP (clonidine and diazepam). For diazepam, there is evidence that it may not only temporarily suppress, but also reverse established late-phase LTP.

## Competing interests

RR, RD, XGL and JS declare that they have no competing interests. OWS declares that he receives research support (independent research grant) from Pfizer, the maker of pregabalin and gabapentin and gives paid lectures for Eurocept, distributor of S-ketamine.

## Authors' contributions

RR and JS conceived the review. RR wrote the general parts of the basic science sections and made the figures. RR, RD and XGL wrote the specific parts of the basic science sections. RR, OWS and JS wrote the section translational aspects and the conclusions. OWS wrote the sections on human models. All authors read and approved the final version of the manuscript.
